# Development and preliminary evaluation of a computer-assisted assessment tool for Chinese prewriting skills in preschoolers

**DOI:** 10.3389/fpsyg.2026.1793395

**Published:** 2026-05-25

**Authors:** Zhongling Liu, Dan Wu, Shaotong Peng, Chuanfei Dong, Yanyan Huo, Yuanyuan Zhang, Jinmeng Zhang, Wei Zhong, Duo Liu, Jinjin Chen

**Affiliations:** 1 Division of Children’s Health Care, School of Medicine, Shanghai Children’s Hospital, Shanghai Jiao Tong University, Shanghai, China; 2 Department of Cognitive Neuroscience, Donders Institute for Brain, Cognition, and Behaviour, Radboud University Medical Center, Nijmegen, Netherlands; 3 Shanghai Zhongyi Institute of Psychology and Perceptual Science, Shanghai, China; 4 Department of Special Education and Counselling, The Education University of Hong Kong, Hong Kong, Hong Kong SAR, China; 5 Shanghai Children’s Hospital, Dipro Medical Research Center, Shanghai, China

**Keywords:** child, preschool, educational measurement, handwriting, psychometrics, psychomotor performance

## Abstract

**Introduction:**

Writing readiness is a critical milestone for children transitioning from preschool to formal schooling. This preliminary study introduces the Chinese Pre-writing Assessment Tool (CPAT), a computer-assisted instrument designed to provide an objective evaluation of pre-writing skills. Unlike traditional measures, the CPAT uniquely integrates both perceptual-motor and linguistic-cognitive components tailored to the Chinese writing system.

**Methods:**

A preliminary evaluation was conducted with 143 senior kindergarteners to examine the tool’s psychometric properties. The analysis assessed internal consistency, test-retest reliability, and inter-rater reliability. Structural Equation Modeling (SEM) was employed to validate the construct validity and explore the predictive relationships between pre-writing components and writing outcomes.

**Results:**

The CPAT demonstrated promising reliability and robust internal structural validity, offering an option for the objective evaluation of pre-writing skills. SEM results indicated that writing legibility is primarily predicted by visual-motor integration (VMI) and orthographic awareness (OA), whereas writing fluency is significantly influenced by VMI speed. Notably, pencil grip emerged as a potential facilitator for Chinese writing performance, showing a distinct pattern compared to alphabetic scripts.

**Discussion:**

These preliminary findings suggest the CPAT is a promising framework for evaluating Chinese pre-writing. However, the small sample size and cross-sectional design limit generalizability. Future work should focus on establishing formal cut-off scores, implementing automated scoring, and incorporating real-time force tracking to enhance the tool’s clinical and predictive utility.

## 1 Background

### 1.1 Challenges of Chinese handwriting acquisition in school-aged children

Due to the diversity of Chinese character strokes and their inherent visual complexity, subtle differences in morphology directly determine the meaning and pronunciation of Chinese characters (Liu and Liu, 2020). Consequently, Chinese handwriting necessitates a high level of precision. Since handwriting poses a significant challenge for students upon elementary school entry, fostering writing readiness is a crucial focus during the transition from preschool to formal education.

Observations indicate that children who transition smoothly into formal schooling often possess specific abilities, such as fine motor coordination, visual-motor integration and orthographic coding, that facilitate the acquisition of Chinese handwriting (van Hartingsveldt et al., 2015). These abilities are collectively termed “pre-writing skills,” and their proficiency significantly predicts the development of future handwriting competence.

Research indicates that the prevalence of handwriting difficulties globally ranges from 5% to 33% (Overvelde and Hulstijn, 2011), specifically within the Chinese context, this figure is estimated to be between 16% and 25% (Jiang and Liu, 2019). School-aged children with writing difficulties often struggle to acquire new academic knowledge due to pre-writing skill deficits (Tse et al., 2014, 2019). Furthermore, an inadequate foundation in pre-writing skills complicates later remediation, potentially leading to cumulative psychological issues such as diminished self-esteem and school aversion (Feder and Majnemer, 2007).

Therefore, the systematic assessment of pre-writing skills in preschool is essential. This necessity is particularly pronounced in China, where the lack of standardized diagnostic tools for developmental dysgraphia prompts clinicians to depend on subjective criteria, such as DSM-5 (Yongfei et al., 2025; Zebo et al., 2026). In light of these diagnostic challenges in later stages, developing a reliable preschool-level assessment of pre-writing skills is crucial for early preventive intervention supporting children at risk of handwriting difficulties.

### 1.2 Pre-writing skills underpinning Chinese character handwriting

Previous studies suggest that handwriting performance relies on a complex interplay of motor and perceptual components, including fine motor control, visual-motor integration (VMI), visual perception, kinesthesia, and sustained attention (Feder and Majnemer, 2007).

Berninger et al. (1992) proposed that early writing development in school-aged children emerges from the interaction between perceptual-motor components (e.g., VMI and fine motor control) and linguistic components (e.g., orthographic-phonological coding and orthographic knowledge) (Berninger et al., 1992). Guided by this theoretical framework, children gradually develop foundational skills during preschool stage which provide a scaffold for formal writing instruction (Yin and McBride, 2015). In the perceptual-motor domain, preschoolers develop basic abilities such as pencil grip and line drawing (van Hartingsveldt et al., 2015). Regarding linguistic components, orthographic awareness (OA) is widely recognized as a pivotal factor.

The OA included two parts of awareness in Chinese’s: awareness of legal spatial configurations, which mainly developed during preschool years, and component knowledge, which matures during elementary school (Ho et al., 2003; Liu et al., 2024). Previous research has consistently demonstrated that radical knowledge significantly influences writing outcomes in school-aged children (Georgiou et al., 2016; Tong et al., 2009). As the visual complexity of Chinese characters places high demands on spatial arrangement, which is a crucial determinant of legibility (Zhang et al., 2023), we hypothesize that the awareness of legal spatial configurations developed during the preschool period serves as a critical precursor for Chinese handwriting acquisition.

Studies indicate that while preschoolers may not fully grasp the complex rules of radical combination, they do have established a basic awareness of legal spatial configurations of Chinese characters prior to school entry (Lin et al., 2019; Qian et al., 2015). By the first year of kindergarten, children typically demonstrate an understanding of the distinctive features of the Chinese writing system, enabling kids to differentiate Chinese logographic characters from alphabetic letters (e.g., English) and non-linguistic illustrations. Following this initial awareness, older preschoolers can accurately identify legal radicals and invalid characters (e.g., those with rotated or missing radicals) (Qian et al., 2015).

Therefore, the simultaneous assessment of both perceptual-motor and linguistic-cognitive components represents a meaningful endeavor in evaluating the pre-writing skills of preschool children.

### 1.3 Assessment of awareness of legal spatial configurations in preschoolers

It is widely acknowledged that modern Chinese characters have undergone extensive simplification, resulting in scripts that may appear overly abstract to young children during their nascent stages of literacy, thereby complicating character memorization. In contrast, Oracle Bone Script (OBS) retains the vivid, pictographic essence of original characters while inherently embodying the legal spatial configurations and component knowledge central to the Chinese writing system (Ottaviano and Ferrara, 2026). Consequently, OBS has been advocated as a viable tool for interventions aimed at enhancing orthographic awareness in school-aged children (Yongfei et al., 2025). By implementing a pairing task involving OBS and their modern counterparts, children are required to align radical components with legal spatial arrangements based on structural layout features. This approach facilitates a targeted evaluation of their awareness of legal spatial configurations while maintaining the children’s interest.

### 1.4 Computerized assessment of handwriting

Handwriting assessment typically focuses on two primary dimensions: legibility and speed. The integration of computer technology has significantly enhanced the quantitative evaluation of these dimensions. By utilizing electronic tablets, researchers can capture precise kinematic data—such as pen tip trajectory, pressure, and velocity—to objectively evaluate the writing skills of school-aged children. Prominent examples include the Chinese Handwriting Analysis System (CHAS) (Li-Tsang et al., 2013) the Smart Handwriting Analysis Recognition Platform (SHARP) (Li-Tsang et al., 2022), and the computerized Minnesota Handwriting Assessment (MHA) for English (Falk et al., 2011).

Beyond character writing, studies have also investigated children’s line and shape drawing capabilities. Objective metrics derived from visual-motor integration tracing tasks serve as effective proxies for fine motor development (Khalid et al., 2010; Lin et al., 2015). Similarly, recent research has analyzed the gestural characteristics of children creating pre-scriptural symbols (Vaivre-Douret et al., 2021), indicating the practicality of using simple line drawings to infer writing readiness.

However, existing computerized tools are predominantly tailored for school-aged children who have already acquired basic writing skills. There remains a scarcity of systematic, objective instruments specifically designed to assess pre-writing skills in preschoolers. Quantitative assessment is critical, as it allows for the granular evaluation of specific deficits, thereby guiding targeted interventions and promoting pre-writing development.

### 1.5 Aim

This study aims to develop and preliminarily evaluate a computer-assessment tool for Chinese prewriting skills in preschoolers. This tool integrates both the perceptual-motor and linguistic-cognitive components influencing skill development. The study also aimed to preliminarily validate the psychometric properties (reliability and validity) of the tool and to investigate the mechanisms by which motor and cognitive factors influence early writing performance.

## 2 Materials and methods

### 2.1 Participants

The participant’s recruitment was conducted in two phases, recruiting a total of 191 preschool children. The total sample included 87 boys (mean age = 75.1 ± 4.49 months) and 104 girls (mean age = 73.9 ± 4.23 months). The initial pilot phase (Phase 1) involved 48 participants (22 boys, 26 girls) from urban regions. The primary validation phase (Phase 2) recruited 143 participants (65 boys, 78 girls), stratified by urban-rural classification: 120 from urban areas, 13 from townships, and 10 from rural areas.

The participants in Phase 2 were recruited using a stratified sampling approach based on the 2020 Shanghai Census data for children aged 3–6 years. The urban-to-rural ratio in our sample (approximately 84:9:7 for city, town and countryside) reflects the actual demographic distribution of preschool-age children in Shanghai.

Prior to participation, oral assent was obtained from each child, and written informed consent was secured from at least one parent or legal guardian. This study was approved by the Ethics Committee of Shanghai Children’s Hospital (2024R053-F01).

Participants were included if they met the following criteria: (1) typically developing students in the final year of kindergarten (Senior Kindergarten/K3); (2) Mandarin Chinese was the primary language spoken at home; and (3) ability to comprehend instructions and cooperate in simple tasks.

Participants were excluded if they presented with: (1) uncorrected visual or hearing impairments, or significant motor impairments; or (2) diagnosed neurological or neurodevelopmental disorders, such as cerebral palsy, autism spectrum disorder, developmental delay, or speech/language disorders.

The individual socioeconomic data were not collected in the study, but neighborhood housing prices within 1 km of each kindergarten were documented as a proxy indicator of local socioeconomic context. Housing prices ranged from approximately 15,000 to 98,000 CNY/m^2^ across sampling sites. The housing prices and SES level of participating kindergarten were documented in Supplementary Appendix Table A.

### 2.2 Procedure

The study utilized a three-phase design: (1) Tool Development, (2) Pilot Experiment and Revision, and (3) Psychometric Validation.

#### 2.2.1 Phase 1: development of the assessment tool

The Chinese Prewriting Assessment Tool (CPAT) targets children in senior kindergarten, a critical preparatory stage for the transition to elementary school handwriting. The development team comprised a multidisciplinary panel of pediatricians, preschool educators, and researchers specializing in child rehabilitation, behavioral development, and educational psychology.

Based on a comprehensive literature review, the team designed the initial framework. Notably, sitting posture was excluded as an independent assessment item, as children at this stage typically demonstrate proficiency in maintaining posture (e.g., stabilizing paper with the non-dominant hand) during writing tasks.

The preliminary CPAT framework consisted of four components:

(1) Fine motor and hand strength: Pinch strength, pencil grip, and writing pressure.

(2) VMI: A line-drawing task assessing velocity, pause duration, and accuracy performance.

(3) Literacy-related cognitive abilities: OA and RAN tasks.

(4) Chinese character writing: A word-copying task assessing velocity, pause duration, and legibility performance.

#### 2.2.2 Phase 2: pilot experiment and revision

The pilot study included 48 participants (22 boys, mean age = 72.0 ± 4.02 months; 26 girls, mean age = 71.6 ± 4.03 months). Assessments were administered on-site by full-time practitioners with over 3 years of experience in child healthcare, who had received standardized training. All subtests were administered sequentially using standardized instructions, with data recorded immediately by the examiners.

Based on pilot feedback, modifications were made to the layout of the test protocols for the VMI and Chinese character writing to clearly distinguish between instruction and response areas. Furthermore, correlation analyses were conducted; subtests exhibiting weak correlations with Chinese character writing performance were removed from the scoring framework but retained as potential covariates for mechanistic analysis.

#### 2.2.3 Phase 3: validation of reliability and validity

The main study engaged 143 participants (65 boys, mean age = 75.9 ± 4.39 months; 78 girls, mean age = 74.9 ± 4.15 months) to evaluate the psychometric properties of the CPAT. Data collection procedures mirrored those of the pilot phase.

Reliability was assessed via internal consistency, test-retest reliability and inter-rater reliability (evaluated using 20 randomly selected samples). Validity was established through the examination of content validity and construct validity.

### 2.3 Instruments and measures

#### 2.3.1 Apparatus

**FIGURE 1:**
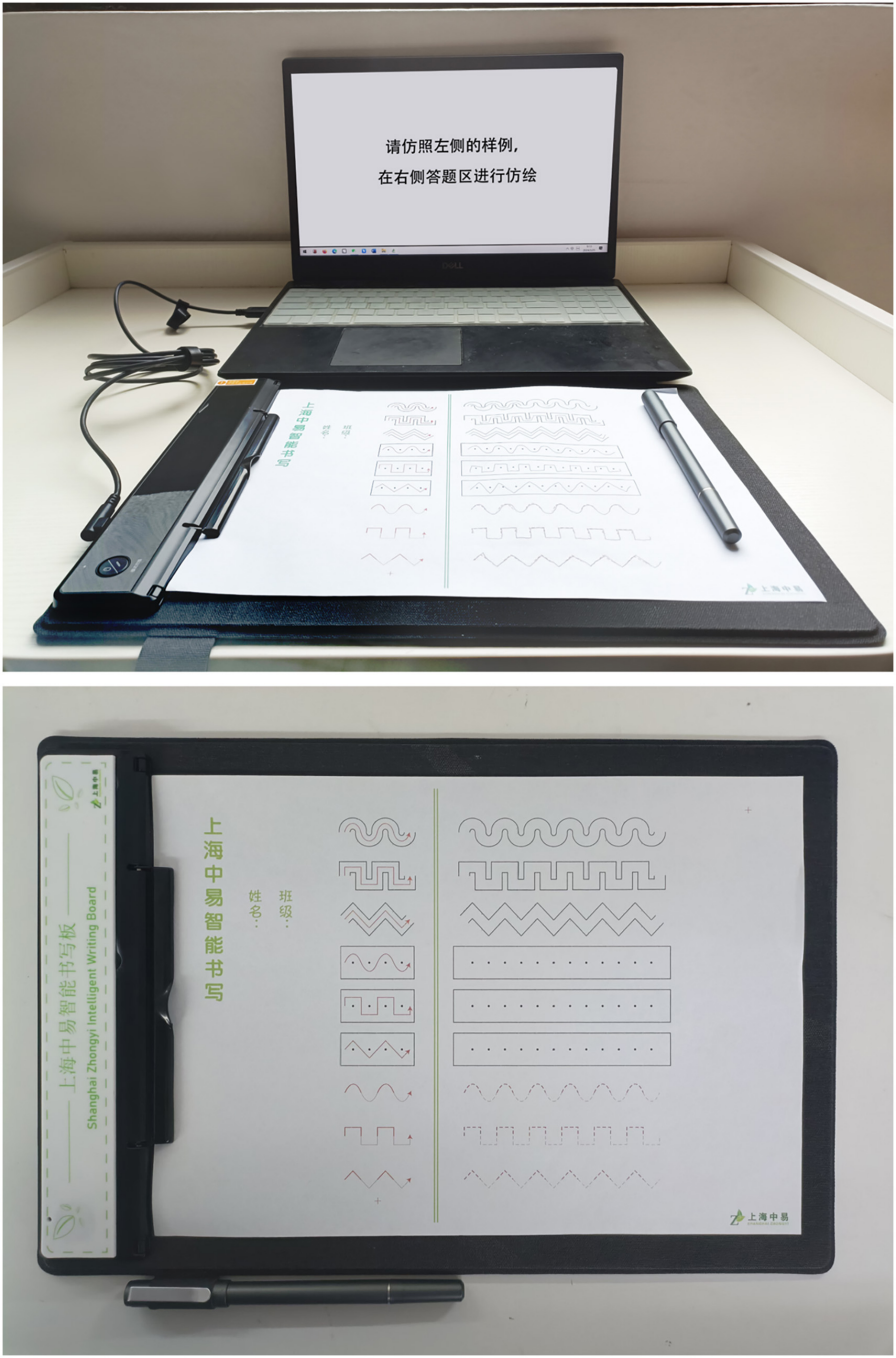
The electronic writing tablets used for CPAT.

The Line Drawing and Word Copying tasks were administered using a Hanvon UGEE (Type HU-A41) electronic handwriting tablet connected to a computer (Figure 1).

(1) Tablet specifications: The device features a 258 × 380 mm surface with a recessed A4 writing area to stabilize the paper.

(2) Stylus: An electromagnetic pen (154 mm length, 11.7 mm diameter, 12.8 g) with a ballpoint tip was used, supporting 2,048 levels of pressure sensitivity.

(3) Data processing: The system records x- and y-coordinates and pressure data at a sampling rate of 200 Hz (every 5 ms). Zero-pressure points were defined as “in-air” movements, while non-zero pressure points indicated active writing. The spatial accuracy was ±0.254 mm. Raw data were uploaded via USB for the extraction of kinematic indicators (trajectory length, duration, pressure) used in the CPAT.

#### 2.3.2 The Chinese Prewriting Assessment Tool (CPAT)

**FIGURE 2:**
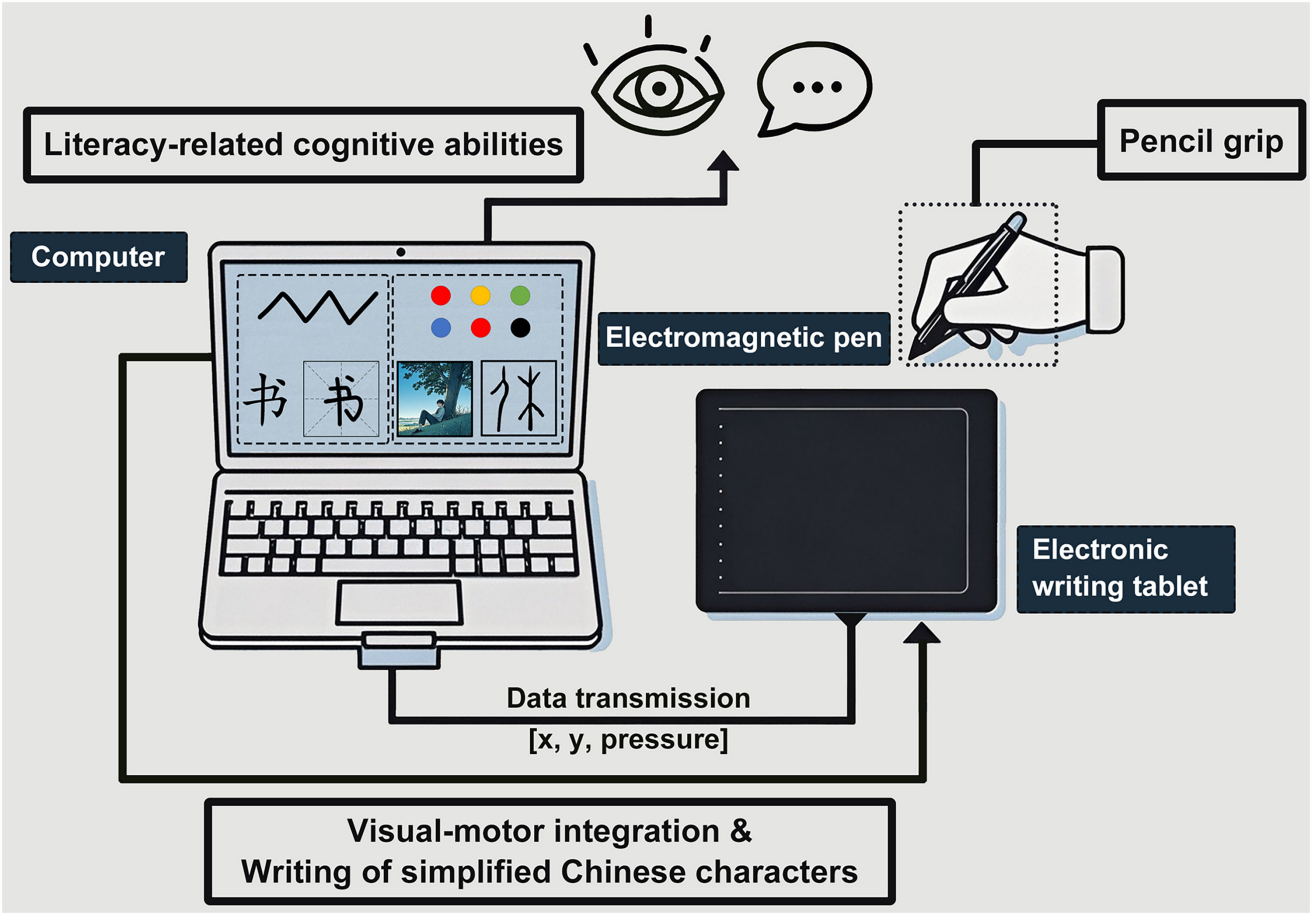
The structural framework of CPAT.

The CPAT was developed to evaluate pre-writing readiness across four domains: (1) Motor: Pencil Grip Assessment; (2) VMI: Line Drawing Task; (3) Literacy-related cognitive abilities: Orthographic Awareness Task and Rapid Automatized Naming Task; and (4) Chinese character writing: Word Copying Task. The structural framework of the CPAT is illustrated in Figure 2.

##### 2.3.2.1 Pencil grip assessment

Pencil grip maturation typically follows a predictable developmental trajectory, categorized into three primary phases (Edwards, 2018): (1) Primitive Phase (1–3 years): Characterized by power-type grasps, such as the *palmar supinate* (1–1.5 years) and *digital pronate* (2–3 years) grasps; (2) Transitional Phase (3–4 years): Emergence of *static tripod* or *quadrupod* grasps, where movement originates from the wrist or elbow rather than isolated finger movements; (3) Mature Phase (4–6 years): Stabilization of the *dynamic tripod* grasp, which facilitates precise distal control through the intrinsic hand muscles.

In this study, pencil grip was operationalized on a four-point scale by trained assessors:

1. 4 points (Mature): Dynamic tripod grip. This “gold standard” pattern utilizes intrinsic hand muscles for fine distal control, allowing coordinated finger movements independent of the arm and trunk.
2. 3 points (Efficient and Transitional): Includes *lateral tripod*, *lateral quadrupod*, and *dynamic quadrupod* grasps. While functional, these patterns are often less ergonomic and more prone to muscular fatigue than the dynamic tripod. This category also encompasses the typical transitional grasp patterns, including *static tripod* or *quadrupod* grasps.
3. 2 points (Inefficient): Intermediate or non-prevalent patterns, such as the *thumb wrap*, *thumb tuck*, *lateral pinch*, or *index grip*.
4. 1 point (Primitive): Immature patterns (e.g., *radial cross palmar*, *palmar supinate*, or *digital pronate*). These involve holding the writing tool in a power-type palmar grasp, relying on gross arm movements for execution.

##### 2.3.2.2 Visual-motor integration (VMI): line drawing task

This task required participants to replicate sample lines using an electronic handwriting tablet without interruption (See the sample in Supplementary Appendix B.1). The task comprised three conditions (totaling nine items: curved, wall, and broken lines), as detailed below:

1. Channel Lines (Stability): Participants traced a path between two solid black lines without touching the borders.
2. Dot avoidance (Coordination): Participants drew lines around obstructing black dots, requiring spatial planning.
3. Line tracing (Force Control): Participants traced over a line, increasing pen pressure when the visual line thickness increased.

This design draws upon the Berry Visual-Motor Integration Test (Crotty and Baron, 2011) and the Mazes subtest of the WIPPSI-R (Pizer and ElBassiouny, 2020).

*Scoring:*

1. Objective metrics: The digitizer recorded total duration (s), writing time (s), pause duration (s), and trajectory length (m). From these, route velocity (cm/s) and pause duration (s) were derived.
2. Accuracy performance: Assessors rated each item on a 4-point scale based on stability, coordination, and force control (total score: 36 points), and the total was identified as performance score.

##### 2.3.2.3 Literacy-related cognitive abilities

1. Orthographic awareness (OA): Detailed in Supplementary Appendix B.2, this task assessed the awareness of legal spatial configuration. It utilized five Oracle Bone Script characters (ancient pictographic antecedents of Chinese characters) that are structurally similar to modern compounds (left-right or top-bottom structures) but unfamiliar to preschoolers (Lin et al., 2019; Yao, 2020). The samples of the practice and formal test part were showed in Supplementary Appendices B.2.1, B.2.2. This approach minimizes rote memory effects while testing the child’s ability to identify pictographic features and radical positions. Three scores were calculated: *OBS_Character* (recognition of characters), *Pic_Character* (matching to image), and *Awareness Development* (structural understanding).
2. Rapid Automatized Naming (RAN): To accommodate preschoolers, a color-naming task was used. A 5 × 4 matrix featuring five randomized colors (red, yellow, green, blue, black) was presented. Following a practice trial to ensure color knowledge, participants were instructed to name the colors in sequence as quickly as possible. The score was the total time (s) taken to complete the matrix (rounded to two decimal places).

##### 2.3.2.4 Chinese character writing: word copying task

Participants copied five two-character words (10 characters total) listed in increasing order of spatial complexity: *Door* (门口), *Autumn* (秋天), *Flower Bed* (花丛), *Forest* (森林), and *Schoolbag* (书包). These items were selected from the Developmental Dyslexia Scale for Standard Mandarin (DDSSM) (Liu et al., 2024) and cover common structures (e.g., single-component, left-right, semi-enclosed) (see Supplementary Appendix B.3).

1. Objective metrics: Kinematic data from the tablet were used to calculate writing velocity (characters/min) and pause duration (s).
2. Legibility performance: A subjective evaluation was assessed via a subjective evaluation framework adapted from the Chinese and English Handwriting Screening Test for Kindergarten Children (CHEST) (Tse et al., 2018). Each character was evaluated across four dimensions: Completeness (stroke integrity), Penmanship (stroke smoothness), Stroke positioning, and Spatial layout (within the grid). Each character received a score out of 4 (Total Performance Score: 40 points). The description and examples on the scoring criteria were in Table 1.

**TABLE 1:** Description and examples on the scoring criteria of Chinese character writing.

Criteria	Reference to the criteria in CHEST	Example (0- no credit; 1- credit)	Description
Completeness	Stroke formation	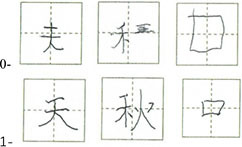	Reflecting the structural integrity by identifying stroke omissions, redundancies, or malformations (e.g., overshooting or incorrect stroke direction).
Penmanship	Not involved	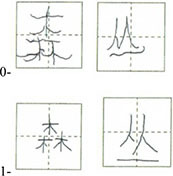	Reflecting the stroke fluency by assessing line smoothness. Credit is awarded for fluid execution, whereas visible tremors or jagged lines result in no credit.
Stroke positioning	Proportion between stokes/radicals alignment and spacing	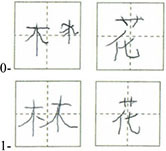	Reflecting the spatial layout of strokes and radicals within the characters, focusing on the appropriate proportion and positioning of radicals according to standard Chinese orthographic rules within a grid.
Spatial layout	Out of grid	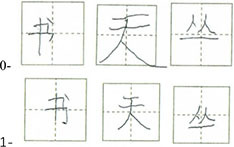	Reflecting the spatial alignment of the character within the grid by identifying if the character is properly centered or significantly offset to one side or exceeds the grid boundaries.

The other assessments were presented in Supplementary Appendix B.4.

### 2.4 Data analysis

Data management and preliminary analyses were performed using Jamovi [Version 2.3.28 (The Jamovi Project, 2022)] and R [R Core Team, 2022]. Structural Equation Modeling (SEM) and Confirmatory Factor Analysis (CFA) were conducted using AMOS 26.0 (IBM Corp, 2014).

#### 2.4.1 Descriptive statistics and group comparisons

All recruited participants successfully completed the assessments; thus, no data were excluded due to missing values. Descriptive statistics are reported as mean ± standard deviation (M±*SD*) for normally distributed variables and median (interquartile range, IQR) for non-normally distributed variables. The normality of data distributions was assessed via visual inspection of histograms and skewness/kurtosis metrics.

Two group differences for continuous variables were evaluated using independent samples *t*-tests or Mann-Whitney U tests, depending on the distribution of the data. To quantify the magnitude of differences, Cohen’s *d* was calculated for *t*-tests and r ($Z/\sqrt{N}$) was calculated for U tests. Furthermore, to control for the family-wise error rate across the 16 multiple comparisons, a Bonferroni correction was employed, with the significance threshold adjusted to *p* < 0.0031. For comparisons involving more than two groups, one-way analysis of variance (ANOVA) or Kruskal-Wallis H tests were employed, depending on the normality of the data. *Post hoc* comparisons were conducted using the Bonferroni correction to identify specific group differences while controlling for multiple-testing errors.

#### 2.4.2 Standardization of scores

To account for the substantial scale disparities across subtasks (e.g., velocity vs. duration) and to ensure model convergence, raw scores were converted to standardized scaled scores (*Mean* = 10,*SD* = 1.5) to conducting factor analyses using the linear transformation formula: *T* = 10+1.5×*Z*. For time-based metrics (e.g., duration) where lower values indicate better performance, scores were inverted using the formula: *T* = 10+1.5×(−*Z*), ensuring that higher scores consistently reflected superior skill mastery.

#### 2.4.3 Psychometric validation

Test-retest reliability and inter-rater reliability was assessed using the Intraclass Correlation Coefficient (ICC) based on a two-way mixed effects model with absolute agreement (single measures). Additionally, the standard error of measurement (SEM) and minimal detectable change (MDC) were calculated for test-retest reliability to quantify measurement error and establish thresholds for meaningful change (Weir, 2005).

Construct validity was examined via Exploratory Factor Analysis (EFA), Confirmatory Factor Analysis (CFA), and Structural Equation Modeling (SEM). Model fit was evaluated using the following indices and cutoffs, as recommended by Schermelleh-Engel et al. (2003): Chi-square ratio (χ^2^/*df* ≤ 2), Comparative Fit Index (*CFI* ≥ 0.95), Tucker-Lewis Index (*TLI* ≥ 0.90), Incremental Fit Index (*IFI* ≥ 0.90), Goodness-of-Fit Index (*GFI* ≥ 0.90), Root Mean Square Error of Approximation (*RMSEA* < 0.05), and Standardized Root Mean Square Residual (*SRMR* ≤ 0.10). Convergent validity was further assessed using standardized factor loadings, Average Variance Extracted (AVE), and Composite Reliability (CR). Parameters were estimated using the Maximum Likelihood (ML) method. Given the low sample-to-parameter ratio, we further employed a bias-corrected bootstrapping procedure with 2,000 resamples to derive more robust standard errors and 95% confidence intervals (CIs) for all estimates (Preacher and Hayes, 2008). Finally, mediation effects were tested using bias-corrected bootstrapping.

## 3 Results

### 3.1 Pilot study and instrument revision

**FIGURE 3:**
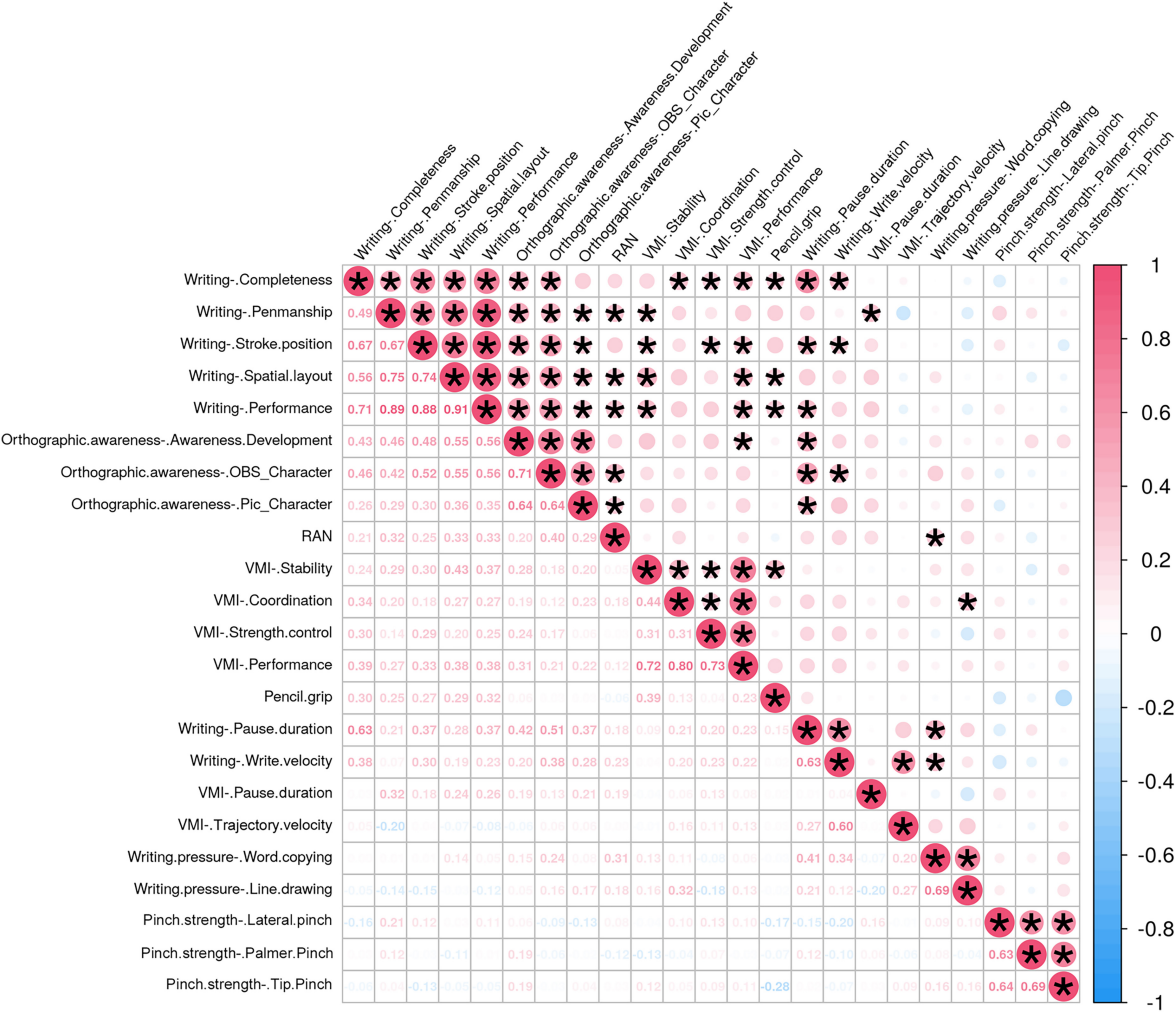
Correlation analysis of the subtests in the initial version of the CPAT. *p < 0.05.

Data from the pilot phase (*N* = 48) were utilized to evaluate and refine the initial CPAT draft (see Supplementary Appendix Table C.1). Pearson correlation analysis revealed that writing pressure and pinch strength were not significantly correlated with either the legibility performance or velocity of Chinese character writing (Figure 3). Consequently, these two metrics were removed from the aggregate scoring framework of the CPAT, although they were retained as auxiliary indicators for mechanistic exploration.

### 3.2 Descriptive statistics and group differences

The main validation study included 143 participants. Raw scores for all subtests are detailed in Supplementary Appendix Table C.2. Generally, no significant sex-based differences were observed across most motor and orthographic awareness subtests. However, after applying the stringent Bonferroni correction, girls significantly outperformed boys in two key qualitative dimensions of Chinese character writing: Penmanship (U = 1811.5, *p* = 0.003, *r* = −0.25) and Stroke Positioning (U = 1786.0, *p* = 0.002, *r* = −0.26). While differences in Pause Duration (*t* = −2.20, *p* = 0.029), Completeness (U = 2046, *p* = 0.030) and Spatial layout (U = 1904, *p* = 0.010) in Chinese character writing were nominally significant at the *p* < 0.05 level, they did not survive the multiple-testing correction.

### 3.3 Reliability analysis

Reliability was assessed via internal consistency and inter-rater reliability. The CPAT demonstrated robust internal consistency, with a McDonald’s ω coefficient of 0.81 (0.79 for boys, 0.82 for girls) and a Cronbach’s α coefficient of 0.80 (0.79 for boys, 0.80 for girls).

Test-retest reliability was focused on RAN and VMI due to their susceptibility to performance fluctuations, whereas Orthographic Awareness and Pencil Grip tasks assess internalized cognitive schemas or ingrained biomechanical habits that typically remain stable over short intervals without targeted intervention (Ho et al., 2003), and word copying is widely recognized paradigm. The CPAT exhibited strong test-retest reliability across its core subtasks. As shown in Supplementary Appendix Table D.1, the ICC values for RAN and VMI components ranged from 0.81 to 0.89, indicating good to excellent temporal stability. The calculated SEM and MDC values further confirmed the precision of these objective metrics.

Inter-rater reliability for the subjective scoring components (accuracy performance in VMI and legibility performance in Chinese character writing) was evaluated by two independent assessors. The ICC indicated excellent agreement, ranging from 0.83 to 0.94 for the VMI and 0.89 to 0.96 for the Chinese character writing (Supplementary Appendix Table D.2).

### 3.4 Validity analysis

#### 3.4.1 Content validity

To ensure the content validity of the CPAT, a panel of five independent external experts was convened. All experts were senior developmental behavioral pediatricians with over 7 years of clinical experience, all holding senior titles. The experts conducted their ratings independently and anonymously to avoid peer influence. During the consultation, some experts suggested incorporating additional dimensions of executive function, such as working memory, to provide a more comprehensive cognitive profile. However, the team decided not to include these metrics after careful consideration, in order to maintain a manageable assessment length and avoid excessive burden on participants, since such functions could be adequately assessed via established tools like the Wechsler Intelligence Scale or other specific cognitive scales. The expert panel ultimately reached a consensus and accepted this rationale, confirming that the current items sufficiently represent the core constructs of Chinese handwriting readiness. The Item-level Content Validity Index (I-CVI) and the Scale-level Content Validity Index (S-CVI/Ave) were 1.00, as detailed in Supplementary Appendix Table E (Polit et al., 2007).

#### 3.4.2 Construct validity

##### 3.4.2.1 Exploratory Factor Analysis (EFA)

Since the CPAT is a newly developed instrument, we first employed EFA to determine the underlying factor structure. EFA was conducted on standardized scores using minimum residual extraction method with oblique rotation, which allows for correlations among factors. The KMO measure of sampling adequacy was 0.78, and Bartlett’s test of sphericity was significant [χ^2^(91) = 689.00, *p* < 0.001].

A three-factor structure was extracted, collectively accounting for 45.4% of the total variance. The model exhibited a robust fit to the data, as indicated by a TLI of 0.941 and an RMSEA of 0.051 (90% CI: 0.014–0.079). The three factors were identified as: (1) Writing Performance, primarily comprising the scores of legibility performance in Chinese character writing; (2) Orthographic Awareness, including the OBS_Character, Pic_Character, and Awareness Development; and (3) Writing Fluency, reflecting the objective metrics in VMI and Chinese character writing. Detailed factor loadings for each item are presented in Supplementary Appendix Table F.1.

##### 3.4.2.2 Confirmatory Factor Analysis (CFA)

**FIGURE 4:**
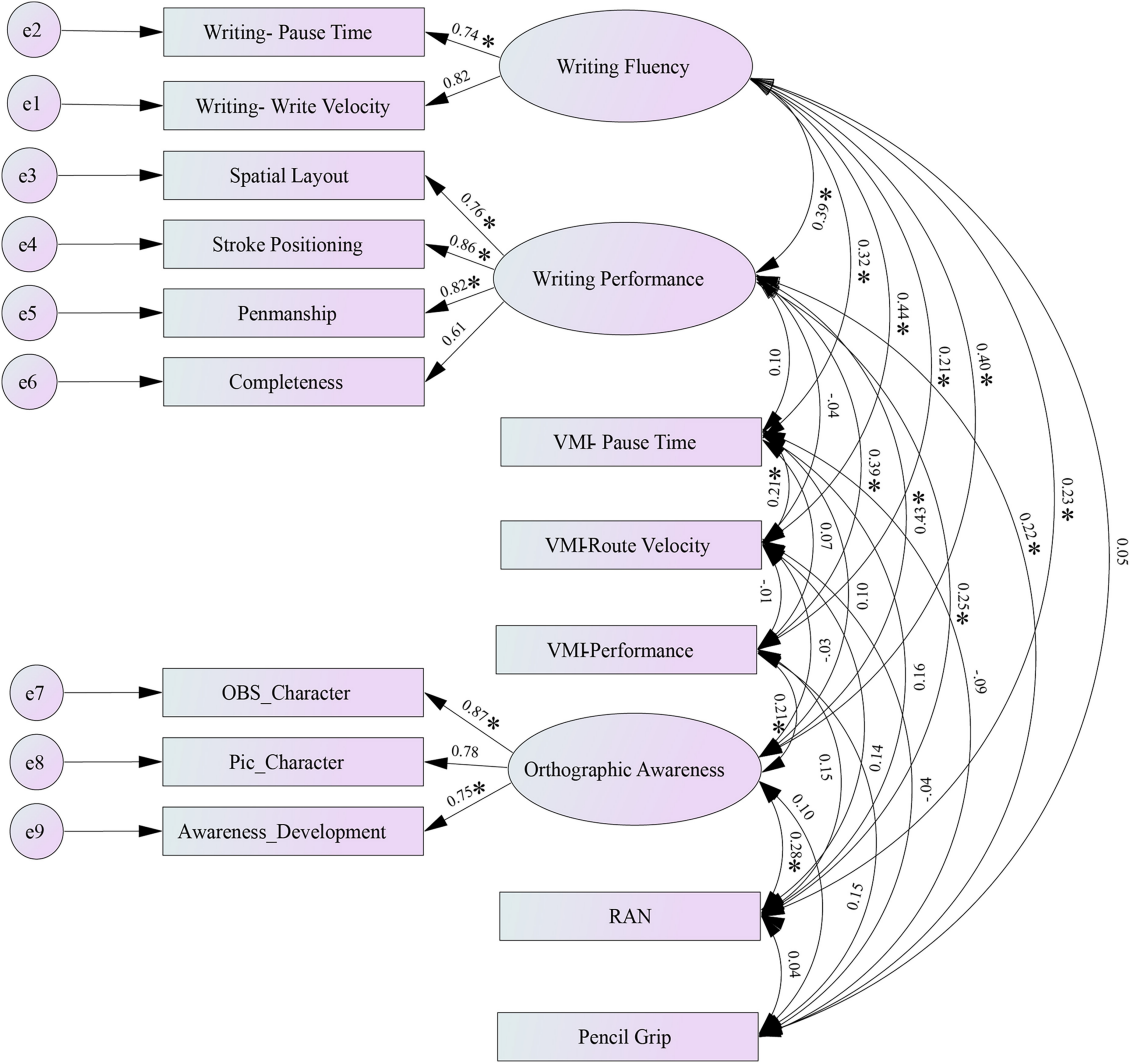
The CFA for the CPAT. *p < 0.05.

Subsequently, CFA was performed to validate the measurement model (Figure 4). Model 1 examined the relationships between latent constructs and their respective observed indicators. All factor loadings were found to be statistically significant (*p* < 0.001), with CR values ranging from 6.88 to 9.66. As detailed in Table 2, the CR and AVE for all constructs met or exceeded the recommended thresholds (CR > 0.7, AVE > 0.5), demonstrating robust convergent validity. Furthermore, the measurement model exhibited a good fit to the data: χ^2^/df = 1.761, CFI = 0.934, GFI = 0.920, RMSEA = 0.073, and SRMR = 0.055.

**TABLE 2:** Measurement model results: factor loadings, reliability, and convergent validity.

Latent constructs	Items	Unstandardized estimate (B)	S.E.	C.R. (*t*-value)	Standardized loading (β)	CR	AVE
Writing fluency	Writing velocity	1.000	–	–	0.819	0.755	0.607
	Pause time	0.468	0.059	7.925***	0.737	–	–
Orthographic awareness	OBS_Character	1.000	–	–	0.868	0.844	0.644
	Pic_Character	0.984	0.102	9.664***	0.784	–	–
	Awareness development	0.686	0.071	9.610***	0.751	–	–
Writing performance	Spatial layout	1.000	–	–	0.757	0.849	0.588
	Completeness	0.852	0.124	6.884***	0.606	–	–
	Penmanship	1.411	0.165	8.528***	0.818	–	–
	Stroke positioning	1.439	0.166	8.647***	0.861	–	–

*N* = 143. ***p < 0.001. S.E., standard error; C.R., critical ratio; β, standardized factor loading; CR, composite reliability; AVE, average variance extracted.

The discriminant validity result met the criteria (see Supplementary Appendix Table F.2).

##### 3.4.2.3 Structural Equation Modeling (SEM)

**FIGURE 5:**
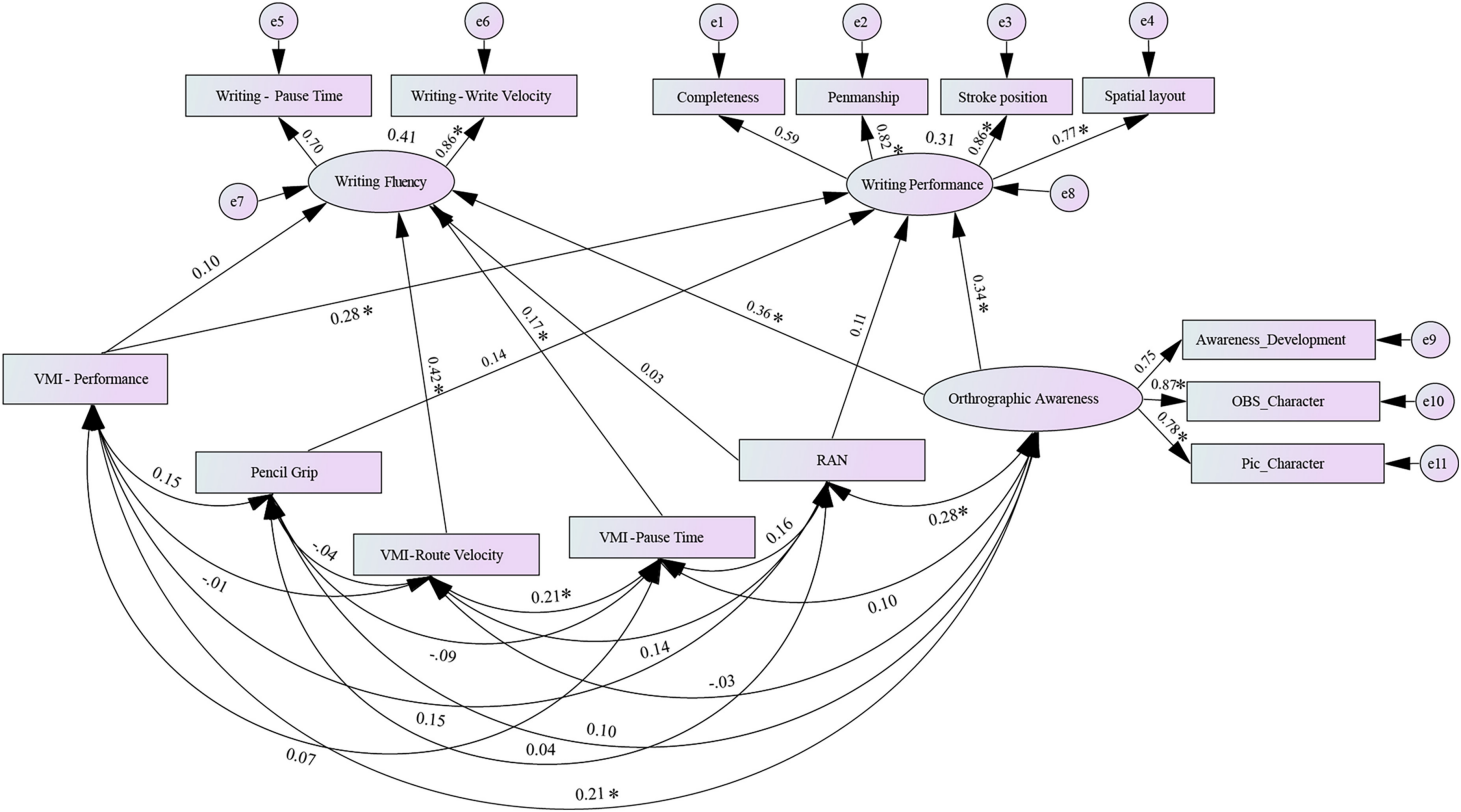
The SEM for the CPAT. *p < 0.05.

Building upon the validated measurement model, a structural model (model 2) was constructed to examine the complex interrelationships between cognitive-motor precursors and handwriting outcomes (Figure 5). The model exhibited a good fit to the data: χ^2^/df = 1.750, RMSEA = 0.073, SRMR = 0.067, GFI = 0.916, CFI = 0.931, and IFI = 0.934.

The structural path analysis revealed that OA served as a robust and balanced predictor for both legibility and fluency of handwriting, exerting significant positive effects on Writing Performance (β = 0.34, *p* < 0.001) and Writing Fluency (β = 0.36, *p* < 0.001). Furthermore, VMI demonstrated differentiated predictive pathways; while the VMI-Performance significantly contributed to Writing Performance (β = 0.28, *p* = 0.002), the VMI-Route Velocity emerged as the strongest direct predictor for Writing Fluency (β = 0.42, *p* < 0.001).

Model stability was validated using a bias-corrected bootstrapping procedure (2,000 resamples). All significant structural paths yielded 95% CIs excluding zero (see Table 3), demonstrating that the findings remain robust and consistent under the current sample size.

**TABLE 3:** Standardized path coefficients and variance explained with bootstrap confidence intervals for Structural Equation Modeling (SEM).

Path/endogenous variable	Unstd. estimate (B)	Std. estimate (β)	Bootstrap 95% CI [BC]	*P*-value (bootstrap)
**Structural paths:**				
→ Writing performance				
OA	0.394	0.340	[0.15, 0.52]	0.001
RAN	0.065	0.109	[−0.07, 0.31]	0.272
Pencil grip	0.129	0.137	[−0.01, 0.28]	0.066
VMI- performance	0.164	0.275	[0.13, 0.43]	0.001
→ Writing fluency				
OA	0.493	0.360	[0.17, 0.56]	0.001
RAN	0.020	0.029	[−0.15, 0.17]	0.740
VMI- performance	0.070	0.099	[−0.09, 0.30]	0.354
VMI- pause time	0.122	0.174	[0.01, 0.37]	0.032
VMI- route velocity	0.297	0.423	[0.22, 0.60]	0.001
**Model explained variance (R^2^):**				
Writing Performance	/	0.314	[0.174, 0.461]	0.005
Writing fluency	/	0.411	[0.171, 0.565]	0.007

*N* = 2,000 bootstrap resamples. Unstd., unstandardized; Std., standardized; BC, bias-corrected.

Collectively, the model explained a substantial proportion of variance in Chinese handwriting readiness, accounting for 31% of the variance in Writing Performance and 41% of the variance in writing velocity.

### 3.5 Inter-correlations of CPAT subtests

**FIGURE 6:**
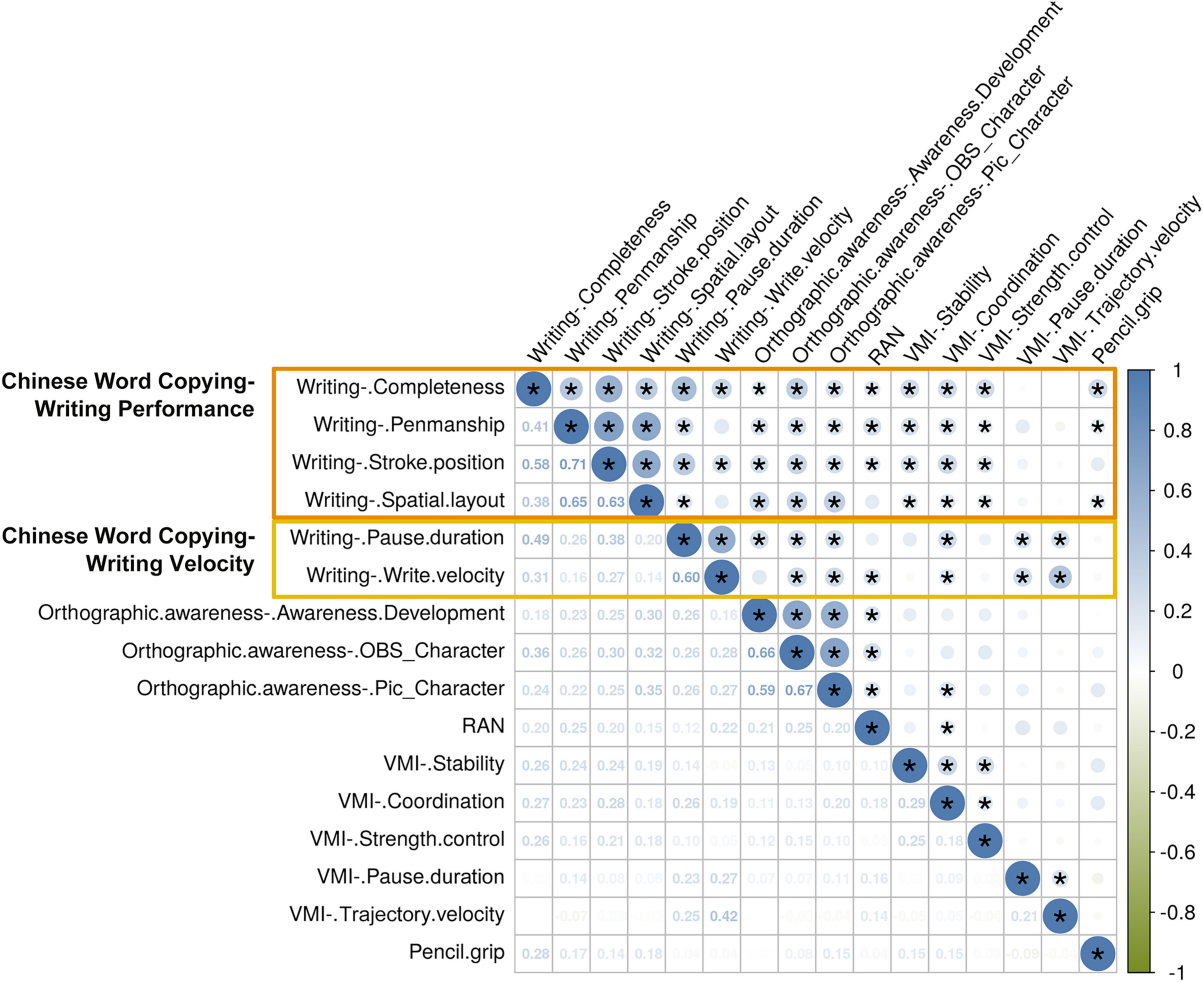
The internal correlation between the subtests of CPAT. *p < 0.05.

Correlation analysis (Figure 6) revealed generally strong internal consistency among the tool’s indicators. Notably, Writing-Performance showed significant positive correlations with OA, RAN, VMI-Performance, and Pencil Grip. Meanwhile, writing velocity was significantly correlated with OA, RAN, and VMI velocity. These findings underscore the multidimensional nature of handwriting readiness during the preschool-to-school transition.

### 3.6 Mechanisms of motor control in handwriting readiness

In the integrated structural model, while Pencil Grip showed a marginally significant positive association with Writing Performance (β = 0.137, *p* = 0.066), its direct impact appeared to be partially suppressed by the inclusion of the overarching VMI-Performance factor. However, to further dissect the specific mechanisms of motor control, a more granular analysis was conducted to explicitly investigate the interrelationships among pencil grip, finger strength, and writing pressure.

**FIGURE 7:**
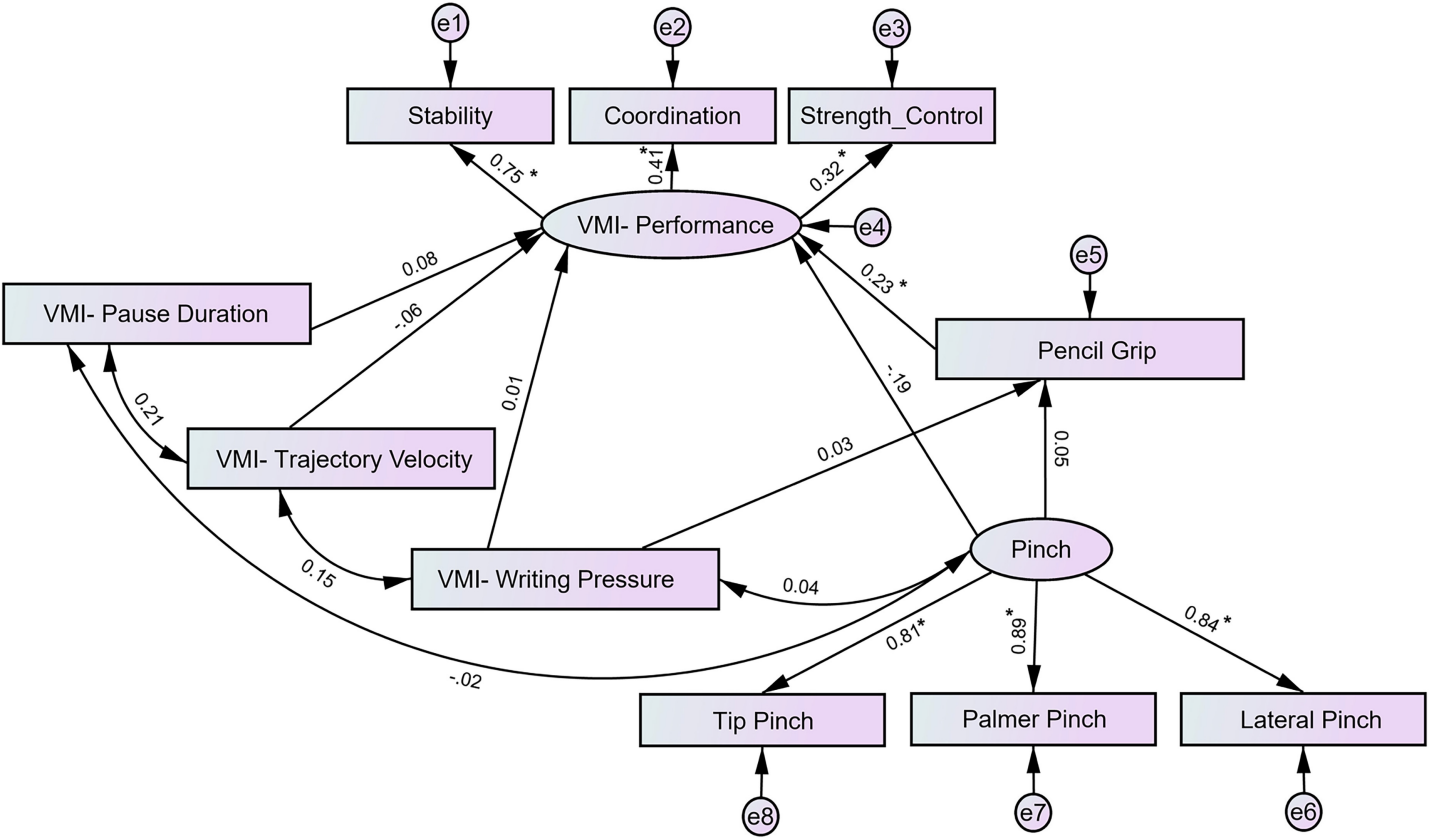
The SEM of strength and motor factors in VMI performance. *p < 0.05.

We constructed a SEM with VMI performance as the endogenous variable (Figure 7). The model demonstrated acceptable fit indices: χ^2^(28) = 43.16, *p* = 0.03, *CFI* = 0.95, *GFI* = 0.94, *RMSEA* = 0.06, and *SRMR* = 0.06.

The SEM results indicated that pencil grip had a significant positive effect on VMI performance (β = 0.23, *p* = 0.033). Conversely, pinch strength showed a negative association with VMI performance, though this effect did not reach statistical significance (β = 0.19, *p* = 0.094).

To further clarify these mechanisms—specifically whether pencil grip mediates the effects of strength factors on VMI performance, or if a suppression effect exists—we conducted a mediation analysis using bias-corrected bootstrapping (Supplementary Appendix Figure G).

The results indicated that pencil grip did not serve as a significant mediator in the relationship between writing pressure and VMI performance (indirect effect: β = 0.005, 95%*CI*[−0.014, 0.046]), nor between pinch strength and VMI performance (indirect effect: β = 0.009, 95%*CI*[−0.013, 0.072]).

Consistent with the SEM findings, the mediation model confirmed that pencil grip exerted a significant direct positive effect on tracing performance (β = 0.265,*p* = 0.042). While pinch strength again showed a negative coefficient (β = −0.176), this direct path remained statistically non-significant (*p* = 0.174).

## 4 Discussion

This preliminary study introduced the CPAT as a potential instrument for assessing pre-writing readiness in Chinese children transitioning from kindergarten to elementary school, integrating perceptual–motor components with literacy-related cognitive dimensions. Our initial findings suggest that the CPAT exhibits encouraging psychometric properties, including acceptable reliability and structural validity, making it a promising tool for objectively evaluating writing readiness in senior kindergarteners.

Through SEM, this study elucidated the specific contributions of perceptual–motor and linguistic-cognitive components to Chinese handwriting legibility performance and fluency. Notably, OA and VMI performance jointly influenced both writing quality and speed, whereas VMI completion velocity emerged as a distinct predictor of writing speed.

### 4.1 Comparative analysis with international and domestic assessment tools

Contemporary trends in preschool handwriting readiness assessment exhibit a shift from unidimensional motor-based approaches toward multidimensional cognitive frameworks. The core conceptual framework of the CPAT developed in this study aligns with internationally recognized instruments, such as the Writing Readiness Inventory Tool In Context (WRITIC) (Haberfehlner et al., 2023) and the Prewriting Assessment (PWA) (Johnston et al., 2025), identifying VMI and fine motor development as critical predictors of future handwriting quality.

Nonetheless, CPAT distinguishes itself through its technical integration and adaptation to the unique characteristics of Chinese orthographic. By utilizing OA tasks to evaluate children’s awareness of legal spatial configuration, CPAT can identify “at-risk” children who may exhibit normal motor function but struggle due to underlying cognitive processing deficits.

Furthermore, the task designs within the CPAT incorporate item formats from established domestic paper-and-pencil instruments, such as the CHEST (Tse et al., 2018) and Handwriting Test for Preschool Children (HT-PRE) (Hong et al., 2020), particularly regarding Chinese character production. The framework also integrates the methodology of stroke-based computerized assessments developed in Taiwan (Li et al., 2018). By leveraging digital tablets to capture kinetic data, the CPAT aims to provide a more precise and objective evidentiary basis for targeted early interventions.

### 4.2 Assessment of data distribution and ceiling effects

A methodological consideration in the current study is the ceiling effect observed in foundational subtasks (see Supplementary Appendix Table C.3), such as Pencil Grip and OA. From a developmental perspective, these results are expected, as older preschoolers typically internalize basic orthographic and motor skills prior to primary school entry. However, the overall utility of our assessment was not compromised; the coefficients of variation across most metrics ensured sufficient variance for our path analyses. These ceiling effects primarily highlight that certain skills have reached a developmental plateau, whereas more nuanced dimensions of handwriting continue to serve as sensitive predictors of individual differences.

### 4.3 Comparability across socioeconomic levels

To evaluate sample comparability across socioeconomic backgrounds, we compared CPAT performance across three SES levels defined by midpoint of the neighborhood housing prices (High SES: ≥55,000 CNY/m^2^, *n* = 84; Meddle SES: 40,000–54,999 CNY/m^2^, *n* = 35; Low SES: <39,999 CNY/m^2^, *n* = 24). The results indicate that participants across different SES levels exhibited comparable performance across the majority of indicators (See Supplementary Appendix Table H). Although significant differences were observed in Coordination in VMI (*p* = 0.047, ε^2^; = 0.043) and OA-OBS_Character (*p* = 0.036, ε^2^ = 0.047), both yielded small effect sizes, suggesting negligible practical differences. The findings suggest that while participants were drawn from diverse socioeconomic backgrounds, their preschool pre-writing performance remained largely consistent. This outcome is plausible given that the study was conducted in Shanghai—a first-tier metropolis where urban-rural disparities are significantly attenuated compared to other regions.

We acknowledge that neighborhood housing price, while an informative proxy, may not fully capture individual-level socioeconomic nuances, such as parental education or household income. Future studies should incorporate direct SES measures to better characterize the sample and examine potential SES-related moderators of prewriting development. Additionally, the sample was drawn exclusively from Shanghai, and findings may not generalize to other regions of China with different demographic compositions.

### 4.4 The role of visual-motor integration (VMI)

Previous research has established a strong association between VMI and handwriting development, often identifying it as a key predictor of school-age writing performance (Hwang et al., 2024; van Hartingsveldt et al., 2015). Our study extends these findings by quantifying the predictive role of VMI on writing velocity using objective digitizer metrics. The CPAT’s VMI task utilizes a constrained line-drawing format rather than traditional shape copying. This design requires children to visually guide their hand movements to ensure the pen tip lands precisely within specific boundaries and changes direction accurately. Given that Chinese characters possess complex spatial structures and low tolerance for stroke errors compared to alphabetic scripts, the ability to control spatial layout in line drawing appears to be a superior predictor of Chinese handwriting competence.

### 4.5 Orthographic awareness and cognitive factors

A distinguishing feature of this study is the incorporation of OA and RAN to capture position awareness, eye movement speed, and orthographic-semantic mapping. Our findings underscore OA as a critical predictor of Chinese character writing in preschoolers. Correlation analysis revealed that performance in matching Oracle Bone Script characters (OBS_Character) was significantly correlated with writing completeness (*r* = 0.36), stroke positioning (*r* = 0.30), and spatial layout (*r* = 0.32). This suggests that children who can identify pictographic and structural patterns are better equipped to apply these insights to handwriting, thereby reproducing character forms with greater structural integrity.

Interestingly, OA showed stronger correlations with “stroke position” and “spatial layout”—dimensions that demand higher cognitive processing. Research on older children with dysgraphia indicates that even when basic character forms are preserved, deficits often manifest as inaccuracies in stroke relative positioning or misalignment within the grid. Our results suggest that during early literacy acquisition, a child’s sensitivity to positional patterns directly scaffolds their ability to manage the spatial layout of Chinese characters. Consequently, targeting OA could be a potent strategy for early intervention.

### 4.6 Interpretation of pause duration in VMI

In the SEM model, pause duration and route velocity in the VMI task loaded differently, preventing their convergence onto a single second-order factor. We speculate that this divergence arises from the relatively low cognitive demand of the VMI task. Unlike the Chinese character copying task, which requires cognitive planning, pauses in the simple VMI task may reflect momentary lapses in attention or motor adjustments rather than cognitive processing time.

### 4.7 The limited role of RAN

While previous studies suggest that RAN influences writing velocity in elementary school children (Cheng-Lai et al., 2013), our SEM results did not identify RAN as a major determinant, despite a significant bivariate correlation. This discrepancy may stem from the developmental stage of the participants. In preschoolers, handwriting has not yet reached the stage of automaticity where visual-verbal access speed (as measured by RAN) becomes a critical bottleneck. Instead, at this early stage, writing readiness is more heavily dictated by orthographic awareness (top-down cognitive processing) and visual-motor integration (bottom-up execution control).

Furthermore, although RAN showed a significant bivariate correlation with writing outcomes, its predictive power was largely absorbed when concurrent cognitive and motor precursors were included in the multivariate model. This suggests that for preschoolers, the integrated coordination of visual and motor skills is a more robust predictor of handwriting readiness than the speed of naming alone.

### 4.8 The facilitative role of pencil grip

A novel finding of this study is the facilitative role of pencil grip in Chinese handwriting. While consistent with conventional pedagogical expectations, this contradicts several studies on alphabetic scripts that found no significant link between grip pattern and legibility (Donica et al., 2018; Schwellnus et al., 2012). We propose two explanations for this discrepancy.

First, the script-dependent hypothesis. Unlike alphabetic letters, Chinese characters are logographic and visually dense, requiring exceptional stroke precision and complex spatial arrangement. A mature pencil grip (e.g., dynamic tripod) provides the necessary stability and distal finger freedom to execute these intricate strokes accurately. In the context of Chinese orthography, the biomechanical efficiency afforded by a mature grip may be more critical for achieving legibility than it is for simpler alphabetic scripts.

Second, the environmental and developmental hypothesis: The observed link might be confounded by home education. In the absence of formal school instruction, preschoolers who receive more parental guidance likely develop both higher OA or familiarity (Ece Demir-Lira et al., 2019) and better pencil grip habits simultaneously. Thus, the association might reflect broader exposure to literacy activities rather than a strictly biomechanical cause. While our supplementary analysis for SES revealed no significant differences in pencil grip across SES levels, we remain cautious in our interpretation given that this is an indirect measure. Consequently, the potential influence of the home literacy environment cannot be entirely ruled out. Furthermore, our data also showed that some children with functional/inefficient grips still achieved good legibility (Supplementary Appendix Figure I), suggesting that while a mature grip is facilitative, it is not the sole determinant of quality.

### 4.9 Mechanisms of grip strength and pressure

Contrary to the assumption that grip patterns are dictated by hand strength, our results indicated that pinch strength and writing pressure had negligible and non-significant effects on pencil grip (β < 0.04, *p* > 0.05). This implies that pencil grip acquisition in this age group may be driven more by motor learning and instruction than by physiological strength maturation.

Furthermore, we observed a non-significant trend where pinch strength had a negative coefficient (β = −0.176) on VMI performance. While statistically inconclusive in this healthy cohort, this trend aligns with findings in writer’s cramp patients (Baur et al., 2009) rather than typical development studies (Engel-Yeger and Rosenblum, 2010). It raises the theoretical possibility that excessive grip force—perhaps due to tension or lack of modulation—might hinder hand flexibility, thereby disrupting fluid visuomotor coordination. Future research with clinical samples is needed to verify this potential “force-flexibility trade-off.”

## 5 Limitations and future directions

Several limitations should be acknowledged. First, as a preliminary psychometric evaluation, this study lacks longitudinal data and external benchmarks to establish formal cut-off scores or predictive utility for the CPAT. Second, the relatively small sample size (*N* = 143) may have limited the statistical power to detect subtle effects, such as those related to pinch strength. Third, the evaluation relied on labor-intensive manual scoring for the legibility dimensions; future iterations should leverage the current dataset to develop automated, objective scoring algorithms.

Furthermore, technical and environmental factors may have influenced the findings. The ballpoint-style electromagnetic pens lack the tactile feedback of traditional pencils, potentially affecting preschoolers’ motor control. Additionally, the measurement of static pinch strength may not fully reflect the dynamic force modulation required during writing. Finally, the absence of direct data on socioeconomic status and home literacy environments means that potential confounding variables were not controlled. Future research should utilize instrumented pens for real-time force tracking and include broader environmental factors to enhance generalizability.

## 6 Conclusion

This study provides preliminary evidence for the development and validation of the Chinese Prewriting Assessment Tool (CPAT), a digital instrument designed to evaluate writing readiness in children transitioning from preschool to school. By integrating perceptual-motor components with literacy-related cognitive dimensions, the CPAT offers a promising framework for capturing a comprehensive profile of early writing capabilities. Initial psychometric testing suggests that the CPAT demonstrates acceptable reliability and validity for early assessment within this specific sample.

Key findings indicate that visual-motor integration (VMI) and orthographic awareness are critical predictors of Chinese handwriting legibility and velocity. Furthermore, preliminary observations suggest that proficient pencil grip might facilitate Chinese writing performance, warranting further investigation to clarify its role relative to findings in alphabetic scripts. These insights highlight the multidimensional nature of handwriting acquisition and point to the potential value of the CPAT for early identification and targeted intervention, though further large-scale longitudinal studies are required to confirm its clinical utility and predictive power.

## Data Availability

The raw data supporting the conclusions of this article will be made available by the authors, without undue reservation.

## References

[B1] Baur B. Fürholzer W. Jasper I. Marquardt C. Hermsdörfer J. (2009). Effects of modified pen grip and handwriting training on writer’s cramp. *Arch Phys Med Rehabil.* 90 867–875. 10.1016/j.apmr.2008.10.015 19406309

[B2] Berninger V. Yates C. Cartwright A. Rutberg J. Remy E. Abbott R. (1992). Lower-level developmental skills in beginning writing. *Reading Writ.* 4 257–280. 10.1007/BF01027151

[B3] Cheng-Lai A. Li-Tsang C. W. Chan A. H. Lo A. G. (2013). Writing to dictation and handwriting performance among Chinese children with dyslexia: Relationships with orthographic knowledge and perceptual-motor skills. *Res. Dev. Disabil.* 34 3372–3383. 10.1016/j.ridd.2013.06.039 23911643

[B4] Crotty K. Baron I. S. (2011). “Beery Developmental test of Visual-motor integration (VMI),” in *Encyclopedia of Clinical Neuropsychology*, eds Kreutzer J. S. DeLuca J. Caplan B. (New York, NY: Springer), 364–365. 10.1007/978-0-387-79948-3_1523

[B5] Donica O. T. Massengill M. Gooden M. J. (2018). A quantitative study on the relationship between grasp and handwriting legibility: Does grasp really matter? *J. Occup. Therapy Schools Early Intervent.* 11 411–425. 10.1080/19411243.2018.1512068

[B6] Ece Demir-Lira O. Applebaum L. R. Goldin-Meadow S. Levine S. C. (2019). Parents’ early book reading to children: Relation to children’s later language and literacy outcomes controlling for other parent language input. *Dev. Sci.* 22:e12764. 10.1111/desc.12764 30325107 PMC6927670

[B7] Edwards S. J. (2018). *Hand Grasps and Manipulation Skills*, 2 Edn, Vol. 7. Milton Park: Routledge.

[B8] Engel-Yeger B. Rosenblum S. (2010). The effects of protracted graphomotor tasks on tripod pinch strength and handwriting performance in children with dysgraphia. *Disabil. Rehabil.* 32 1749–1757. 10.3109/09638281003734375 20373859

[B9] Falk T. H. Tam C. Schellnus H. Chau T. (2011). On the development of a computer-based handwriting assessment tool to objectively quantify handwriting proficiency in children. *Comput. Methods Programs Biomed.* 104 e102–e111. 10.1016/j.cmpb.2010.12.010 21376418

[B10] Feder K. P. Majnemer A. (2007). Handwriting development, competency, and intervention. *Dev. Med. Child Neurol.* 49 312–317. 10.1111/j.1469-8749.2007.00312.x 17376144

[B11] Georgiou G. K. Aro M. Liao C. H. Parrila R. (2016). Modeling the relationship between rapid automatized naming and literacy skills across languages varying in orthographic consistency. *J. Exp. Child Psychol.* 143 48–64. 10.1016/j.jecp.2015.10.017 26615467

[B12] Haberfehlner H. de Vries L. Cup E. H. C. de Groot I. J. M. Nijhuis-van der Sanden M. W. G. (2023). Ready for handwriting? A reference data study on handwriting readiness assessments. *PLoS One* 18:e0282497. 10.1371/journal.pone.0282497 36867627 PMC9983835

[B13] Ho C. S.-H. Ng T.-T. Ng W.-K. (2003). A “Radical” approach to reading development in Chinese: The role of semantic radicals and phonetic radicals. *J. Literacy Res.* 35 849–878. 10.1207/s15548430jlr3503_3 42146917

[B14] Hong Q. Jiang B. Xu Q. Zhang L. Ou J. Zhang Q. et al. (2020). Reliability and validity of Handwriting test for preschool children (HT-PRE): A new tool to assess the handwriting ability of preschool children aged 5-6 years old in Mainland China. *PLoS One* 15:e0229786. 10.1371/journal.pone.0229786 32119715 PMC7051084

[B15] Hwang Y. S. Hsiao Y. L. Su P. F. Hung J. Y. Tsai W. H. (2024). Kindergarten visual-perceptual and motor skills and behavioral traits predict first-Grade Chinese handwriting legibility and speed. *Am. J. Occup. Ther.* 78 7801205170. 10.5014/ajot.2024.050425 38165221

[B16] IBM Corp. (2014). *Amos.* Armonk, NY: IBM Corp.

[B17] Jiang Z. Liu X. (2019). *Operational Treatment Techniques for Child Developmental Disorders.* Beijing: People’s Health Publishing House.

[B18] Johnston B. Levay Z. Ryan B. Hatfield M. Calder S. D. Claessen M. (2025). The concurrent validity and reliability of the Prewriting assessment. *Aust. Occup. Ther. J.* 72:e70059. 10.1111/1440-1630.70059 41383024

[B19] Khalid P. I. Yunus J. Adnan R. (2010). Extraction of dynamic features from hand drawn data for the identification of children with handwriting difficulty. *Res. Dev. Disabil.* 31 256–262. 10.1016/j.ridd.2009.09.009 19854613

[B20] Li C.-H. Wu H.-M. Kuo B.-C. Yang Y.-M. Lin C.-K. Wang W.-H. (2018). The validity of computerized visual motor integration assessment using Chinese basic strokes. *Interactive Learn. Environ.* 26 1074–1089. 10.1080/10494820.2018.1442867

[B21] Lin D. Mo J. Liu Y. Li H. (2019). Developmental changes in the relationship between character reading ability and orthographic awareness in Chinese. *Front. Psychol.* 10:2397. 10.3389/fpsyg.2019.02397 31708837 PMC6824357

[B22] Lin Q. Luo J. Wu Z. Shen F. Sun Z. (2015). Characterization of fine motor development: Dynamic analysis of children’s drawing movements. *Hum. Mov. Sci.* 40 163–175. 10.1016/j.humov.2014.12.010 25574765

[B23] Li-Tsang C. W. P. Wong A. S. K. Leung H. W. H. Cheng J. S. Chiu B. H. W. Tse L. F. L. et al. (2013). Validation of the Chinese handwriting analysis system (CHAS) for primary school students in Hong Kong. *Res. Dev. Disabil.* 34 2872–2883. 10.1016/j.ridd.2013.05.048 23816625

[B24] Li-Tsang C. Li T. M. H. Yang C. N. Leung H. Zhang E. (2022). Evaluating Chinese handwriting performance of primary school students using the Smart handwriting analysis and recognition platform (SHARP). *medRxiv* [Preprint] 10.1101/2022.02.19.22270984

[B25] Liu S. Liu D. (2020). Visual-spatial attention and reading achievement in Hong Kong Chinese children: Evidence from a one-year longitudinal study. *Sci. Stud. Reading* 24 214–228. 10.1080/10888438.2019.1648475

[B26] Liu Z. Wang L. Wang T. Chen L. Dong C. Zhang Y. et al. (2024). The developmental dyslexia scale for standard mandarin: A study among early primary students. *Res. Dev. Disabil.* 154:104841. 10.1016/j.ridd.2024.104841 39306968

[B27] Ottaviano L. Ferrara S. (2026). Mechanisms at the core of the Chinese script invention. *Cogn. Linguistics* 37 39–62. 10.1515/cog-2025-0041 41768398 PMC12949599

[B28] Overvelde A. Hulstijn W. (2011). Handwriting development in grade 2 and grade 3 primary school children with normal, at risk, or dysgraphic characteristics. *Res. Dev. Disabil.* 32 540–548. 10.1016/j.ridd.2010.12.027 21269805

[B29] Pizer J. ElBassiouny A. (2020). “Wechsler preschool and primary scale of intelligence (WPPSI),” in *The Wiley Encyclopedia of Personality and Individual Differences*, eds Carducci B. J. Nave C. S. Mio J. S. Riggio R. E. (Hoboken, NJ: Wiley), 473–475. 10.1002/9781119547167.ch148

[B30] Polit D. F. Beck C. T. Owen S. V. (2007). Is the CVI an acceptable indicator of content validity? Appraisal and recommendations. *Res. Nurs. Health* 30 459–467. 10.1002/nur.20199 17654487

[B31] Preacher K. J. Hayes A. F. (2008). Asymptotic and resampling strategies for assessing and comparing indirect effects in multiple mediator models. *Behav. Res. Methods* 40 879–891. 10.3758/BRM.40.3.879 18697684

[B32] Qian Y. Song Y.-W. Zhao J. Bi H.-Y. (2015). The developmental trend of orthographic awareness in Chinese preschoolers. *Reading Writ.* 28 571–586. 10.1007/s11145-014-9538-8

[B33] R Core Team (2022). *R: A Language and Environment for Statistical Computing.* Vienna: R Foundation for Statistical Computing.

[B34] Schermelleh-Engel K. Moosbrugger H. Müller H. (2003). Evaluating the fit of structural equation models: Tests of significance and descriptive goodness-of-fit measures. *Methods Psychol. Res.* 8 23–74. 10.23668/psycharchives.12784

[B35] Schwellnus H. Carnahan H. Kushki A. Polatajko H. Missiuna C. Chau T. (2012). Effect of pencil grasp on the speed and legibility of handwriting in children. *Am. J. Occup. Therapy* 66 718–726. 10.5014/ajot.2012.004515 23106992

[B36] The Jamovi Project (2022). Available online at: https://www.jamovi.org (accessed July 14, 2025).

[B37] Tong X. McBride-Chang C. Shu H. Wong A. M. Y. (2009). Morphological awareness, orthographic knowledge, and spelling errors: Keys to understanding early chinese literacy acquisition. *Sci. Stud. Read.* 13 426–452. 10.1080/10888430903162910

[B38] Tse L. F. L. Siu A. M. H. Li-Tsang C. W. P. (2018). Screening out Chinese–english biliterate kindergarten children with handwriting difficulties. *J. Occup. Therapy Schools Early Intervent.* 11 426–439. 10.1080/19411243.2018.1523769

[B39] Tse L. F. L. Siu A. M. H. Li-Tsang C. W. P. (2019). Developmental skills between kindergarten children with handwriting difficulties in Chinese and/or English. *Australian Occup. Therapy J.* 66 292–303. 10.1111/1440-1630.12550 30565256

[B40] Tse L. F. L. Thanapalan K. C. Chan C. C. H. (2014). Visual-perceptual-kinesthetic inputs on influencing writing performances in children with handwriting difficulties. *Res. Dev. Disabil.* 35 340–347. 10.1016/j.ridd.2013.11.013 24333804

[B41] Vaivre-Douret L. Lopez C. Dutruel A. Vaivre S. (2021). Phenotyping features in the genesis of pre-scriptural gestures in children to assess handwriting developmental levels. *Sci. Rep.* 11:731. 10.1038/s41598-020-79315-w 33436668 PMC7804314

[B42] van Hartingsveldt M. J. Cup E. H. Hendriks J. C. de Vries L. de Groot I. J. Nijhuis-van et al. (2015). Predictive validity of kindergarten assessments on handwriting readiness. *Res. Dev. Disabil.* 36 114–124. 10.1016/j.ridd.2014.08.014 25462472

[B43] Weir J. P. (2005). Quantifying test-retest reliability using the intraclass correlation coefficient and the SEM. *J. Strength Cond. Res.* 19 231–240. 10.1519/15184.1 15705040

[B44] Yao Y. (2020). *Development of Preschool Children’s Awareness of Chinese Characters.* Master thesis. Guangzhou: Jinan University.

[B45] Yin L. McBride C. (2015). Chinese kindergartners learn to read characters analytically. *Psychol. Sci.* 26 424–432. 10.1177/0956797614567203 25711130

[B46] Yongfei J. Xia C. Xinlin Z. Wei Z. Hong L. Dazhi C. et al. (2025). Expert consensus on the standardized construction of learning difficulties clinics. *Chinese J. Child Health Care* 33 349–354. 10.11852/zgetbjzz2025-0286

[B47] Zebo X. Xiaocong C. Caicai Z. Marieke L. Zhenguang C. (2026). Developmental dysgraphia in Chinese: A review of neurocognitive mechanisms, classification, and assessment. *PsyArXiv* [Preprint] 10.31234/osf.io/v23sk_v1

[B48] Zhang L. Xia Z. Zhao Y. Shu H. Zhang Y. (2023). Recent advances in chinese developmental dyslexia. *Annu. Rev. Linguistics* 9 439–461. 10.1146/annurev-linguistics-030421-065648

