# Supplementary material for: Development and preliminary evaluation of a computer-assisted assessment tool for Chinese prewriting skills in preschoolers
Source: Front Psychol. 2026 May 25;17:1793395. doi: 10.3389/fpsyg.2026.1793395 (PMC13245145; doi:10.3389/fpsyg.2026.1793395)

Supplementary materials

Appendix Table A. The housing prices and SES level of participating kindergarten.

ID	Location	Area Classification	Sample Size	Housing Price Range (CNY/m²)	Housing Price Midpoint (CNY/m²)	SES Level
K1	Qingpu New City	Urban	40	54,000–98,000	76,000	High
K2	Yangpu District	Urban	20	48,000–85,000	66,500	High
K3	Pudong District	Urban	24	48,000–65,000	56,500	High
K4	Jiading District	Urban	15	40,000–50,000	45,000	Middle
K5	Yangpu District	Urban	20	35,000–46,000	40,500	Middle
K6	Jiading District	Township	15	30,000–43,000	36,500	Low
K7	Songjiang District	Rural	9	15,000–25,000	20,000	Low

Note. The SES standards were established based on the reference midpoint of the housing price range: a High SES is defined as ⩾55,000 CNY/m², a Middle SES as 40,000–54,999 CNY/m², and a Low SES as <39,999 CNY/m².

Appendix B.1. The sample for VMI task.

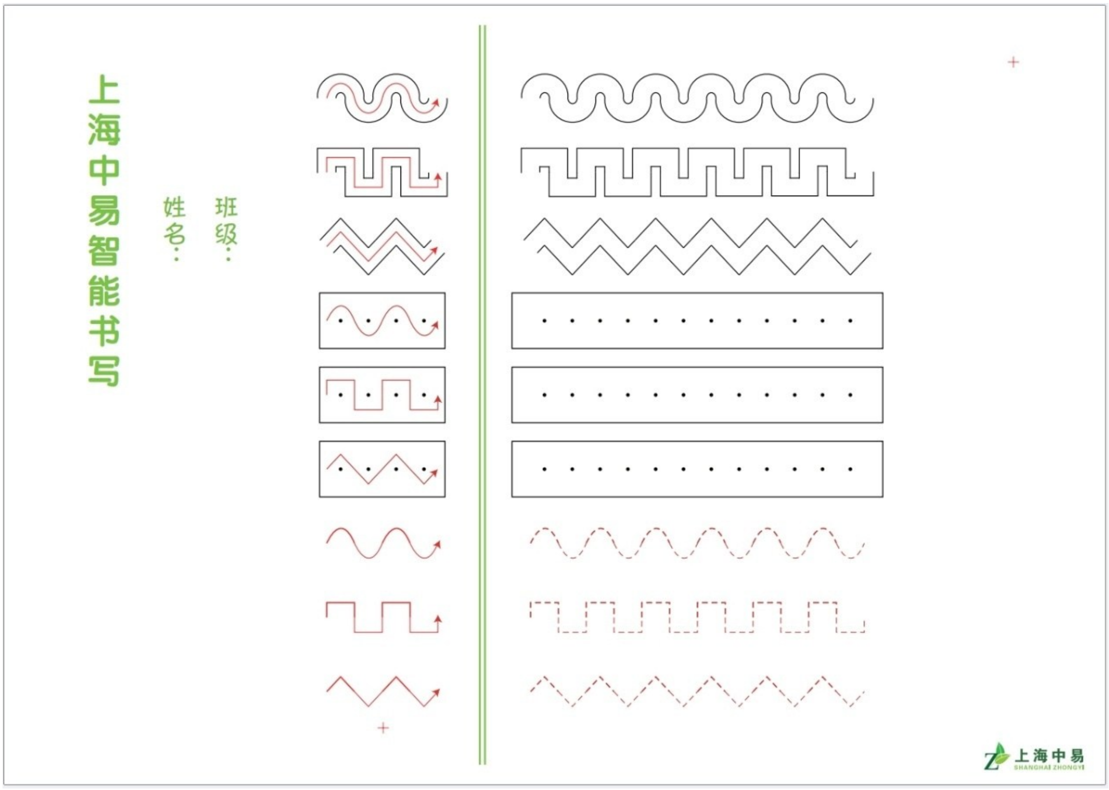

Appendix B.2. Content and Scoring Criteria for Orthographic Awareness

The assessment comprises two phases: a Practice Phase and a Formal Test Phase.

(1) Practice Phase

The purpose of this phase is to familiarize participants with the task requirements and the logic of pictographic evolution. It involves two sequential steps:

1. OBS-Picture Matching: Participants are presented with Oracle Bone Script (OBS) characters and corresponding pictures in a randomized order. They are asked to match each OBS character to its semantically equivalent picture. Upon completion, the assessor displays the correct pairs and briefly explains the pictographic logic underlying the OBS.
2. OBS-Character Matching: Participants are presented with OBS characters and modern Chinese characters. They are asked to match the OBS to the corresponding modern character. Upon completion, the assessor reveals the correct answers and explains the evolutionary process from OBS to modern Chinese characters based on pictographic logic.

Materials: The practice phase includes two trials using familiar, single-component Chinese characters that are widely recognized by kindergarteners. Trial 1 contains three characters, and Trial 2 contains four. These characters were selected for their high pictographic distinctiveness and are arranged from easy to difficult based on visual complexity and frequency of use.

Appendix Figure B.2.1. Samples of practice part in Orthographic Awareness

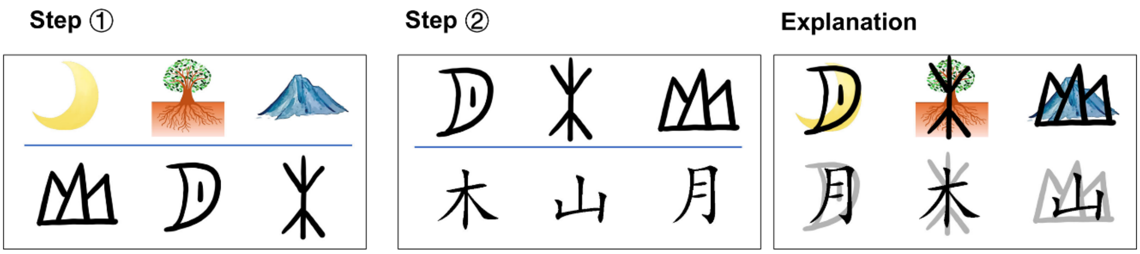

(2) Formal Test Phase

Materials: The formal test utilizes five Chinese characters with strong pictographic features, including compound characters with left-right or top-bottom structures. To evaluate orthographic awareness rather than rote memory, these characters are low-frequency and generally unfamiliar to senior kindergarten children.

Procedure: The formal test consists of three sequential steps (A, B, and C):

- Step A (Baseline Naming): Participants are shown the five target Chinese characters and asked to name them. (Score: 1 point per correct answer).
- Step B (Inference via OBS): Participants are shown correct pairings of OBS and pictures, alongside the target Chinese characters (presented in a randomized order). They are asked to match the OBS/Picture pairs to the corresponding Chinese characters. (Score: 1 point per correct answer).
- Step C (Transfer/Learning Check): Participants are presented with the target Chinese characters and a new set of pictures (semantically identical to Step B but visually distinct). They are asked to match the pictures to the Chinese characters. (Score: 1 point per correct answer).

Appendix Figure B.2.2. Samples of formal test part in *Orthographic Awareness*

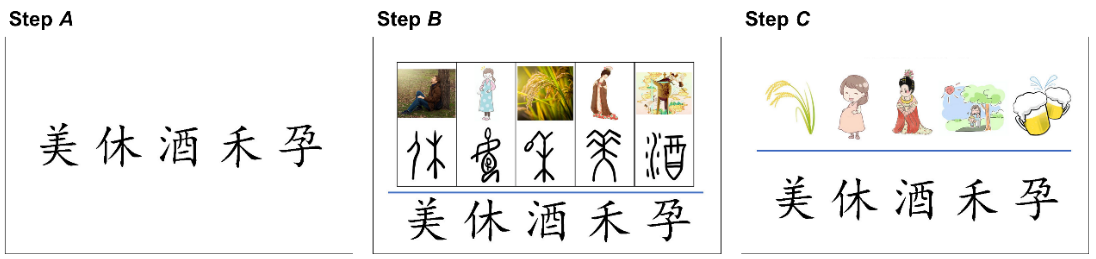

(3) Scoring Criteria

The scoring system yields two types of metrics:

a. Raw Scores:

- OBS_Character: The total score obtained in Step B (Range: 0–5).
- Pic_Character: The total score obtained in Step C (Range: 0–5).

b. Awareness Development Score:

This composite score evaluates the child's ability to use orthographic cues to reason about and acquire new characters. Based on the performance comparison across Steps A, B, and C, children are classified into a 5-point hierarchy (ranging from 1 to 5):

- 5 points (Strong Literacy & Orthographic Awareness): Scores for A, B, and C are all 5 (i.e., perfect performance across all steps).
- 4 points (Acquisition via Awareness): The score for C is higher than A ($C > A$). This indicates the child successfully utilized orthographic awareness during the task to recognize or learn characters they could not name initially.
- 3 points (Scaffolding Dependency): The score for C is less than or equal to A, but the score for B is higher than A ($C \leq A$ and $B > A$). This indicates the child can match characters with the immediate help of OBS prompts but failed to transfer this knowledge to the new picture task (Step C).

- 2 points (No Effect): The scores for A, B, and C are identical and non-zero ($A = B = C \neq 0$). This suggests that the orthographic cues did not alter or improve the child's recognition.
- 1 point (Weak Literacy/Confusion): Any other case, typically where A is the highest score or all scores are zero ($A > B$ or $A = B = C = 0$), indicating a lack of understanding or interference.

Appendix B.3. The sample for Chinese Character Writing task.

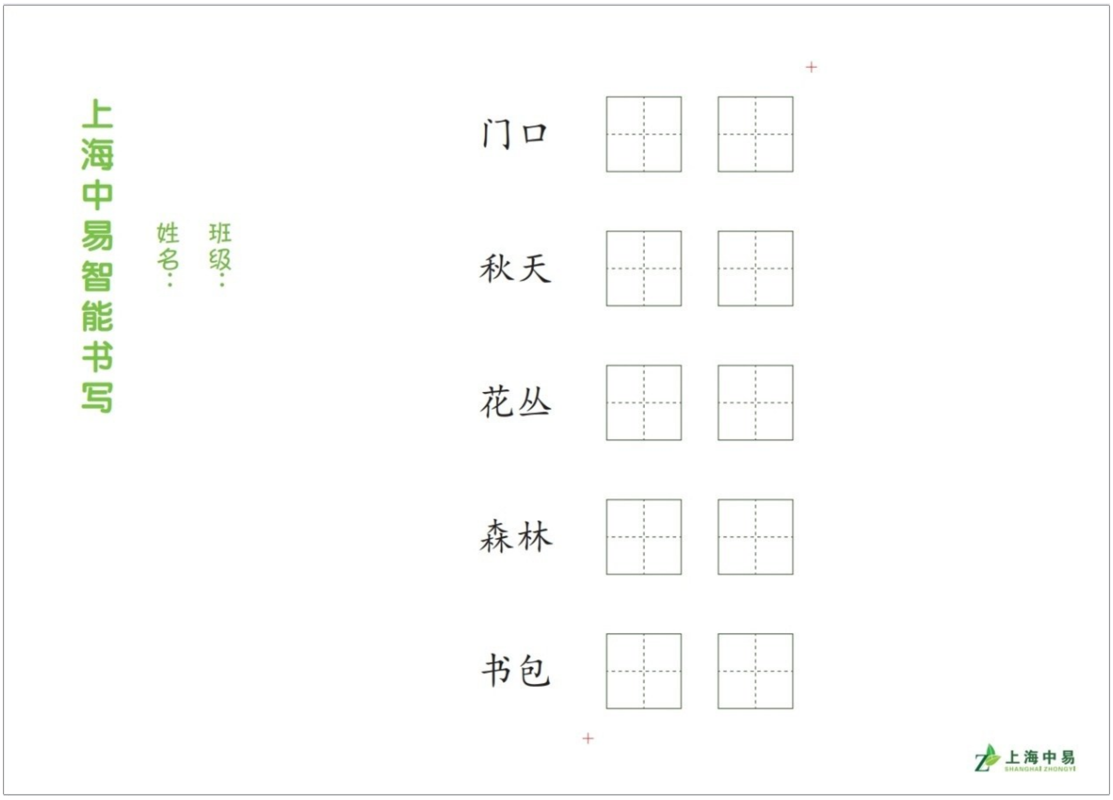

Appendix B.4. The other assessments

Writing Pressure

Writing pressure was quantified as the average pressure level exerted on the stylus tip during the Line Drawing Task, directly recorded by the electronic handwriting tablet.

Pinch strength

Pinch strength was evaluated using three standard grip configurations:

1. Lateral Pinch: Contact between the pad of the thumb and the lateral aspect of the index finger.
2. Palmar Pinch: Contact between the pads of the thumb, index finger, and middle finger.
3. Tip Pinch: Contact between the tips of the thumb and index finger.

Equipment & Procedure:

Assessments were conducted using a ZY332 Electronic Pinch Strength Gauge (Range: 0–100 N; Accuracy: 0.1%; see figure below) connected to a computer interface for real-time data acquisition.

Under the guidance of the assessor, participants positioned their fingers on the gauge according to the specific pinch type. Upon receiving a computerized prompt, participants were instructed to squeeze the gauge with maximum effort, maintain the force for 2 seconds, and then release. Data recording ceased upon release.

Scoring: Each pinch configuration was tested in two trials, separated by a 15-second rest interval to prevent fatigue.

- Trial Score: Calculated as the mean force maintained during the plateau phase (the

interval between reaching peak force and the onset of release).

- Final Score: The arithmetic mean of the two trials for each pinch type, measured in Newtons (N).

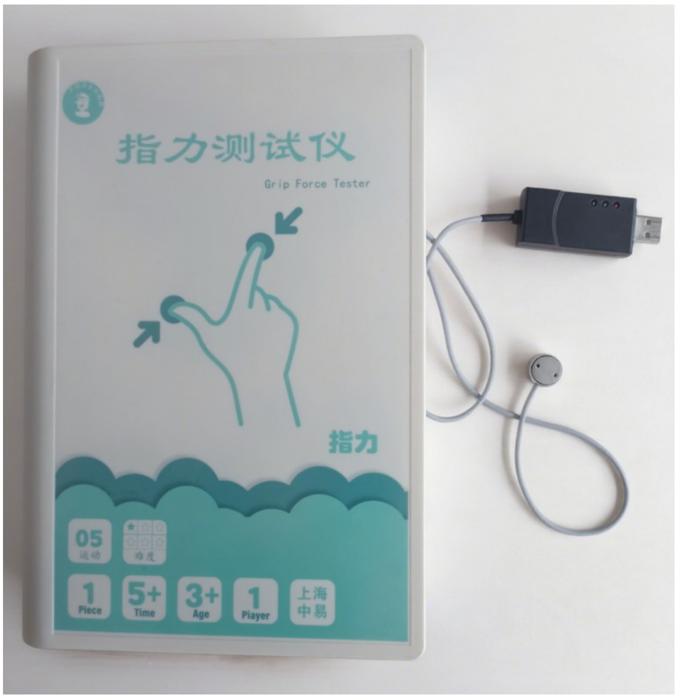

Appendix Table C.1. The results of the initial version of the CPAT in pilot experiment

Items (Raw score)		Male (n=22)	Female (n=26)	*t or U*	*P* value
**Visual-Motor Integration (VMI)**					
**Pause duration (s)**		50.8±22.260	52.1±36.042	0.154	0.88
**Route velocity (cm/s)**		0.73±0.257	0.76±0.439	0.213	0.83
**Performance**					
	Stability	8.45±2.385	9.15±2.034	1.097	0.28
	Coordination	8.00±3.024	7.08±3.085	-1.042	0.30
	Strength control	5.50±2.464	6.23±3.179	0.878	0.39
**Chinese Character Writing**					
**Pause duration (s)**		74.69±44.293	52.56±17.124	-2.352	0.02
**Write velocity (pcs/s)**		0.058±0.022	0.076±0.027	2.387	0.02
**Performance**					
	Completeness	9.00(2.00)	10.00(1.00)	189	0.03
	Penmanship	4.00(8.50)	7.50(7.00)	214	0.13
	Stroke positioning	4.00(3.50)	6.00(3.00)	155	0.01
	Spatial layout	3.50(3.75)	7.00(4.75)	188	0.04
**Literacy-related cognitive abilities**					
**Orthographic awareness**					
	OBS_Character	5.00(3.50)	1.00(3.00)	206	0.07
	Pic_Character	5.00(3.00)	2.75(3.00)	233	0.25
	Awareness Development	4.00(3.25,4.00)	4.00(3.25,4.75)	251	0.43

**RAN (s)**	20.85±6.040	17.96±4.204	-1.946	0.06
**Fine motor skills and hand strength**				
**Pencil grip**	4.00(2.00,4.00)	4.00(3.00,4.00)	253	0.45
**Writing pressure**				
Line drawing task	7581.77±490.879	7660.35±429.766	0.591	0.56
Word copying task	6770.23±771.336	7219.19±480.163	2.460	0.02
**Pinch strength (N)**				
Tip pinch	27.82±7.105	23.92±4.704	-2.273	0.03
Palmar pinch	18.86±4.988	17.07±3.468	-1.464	0.15
Key pinch	18.58±5.230	16.53±3.989	-1.537	0.13

Note. Sensitivity analysis using non-parametric tests yielded consistent results.

Appendix Table C.2. The results of the CPAT in main study

Items (Raw score)		Male (*n*=65)	Female (*n*=78)	*t or U*	*P* value ()	Effect Size (*d or r*)
**Visual-Motor Integration (VMI)**						
**Pause duration (s)**		42.7418.614	47.73±28.810	1.201	0.232	24.709
**Route velocity (cm/s)**		0.8±0.353	0.78±0.316	-0.31	0.757	0.333
**Performance**		22.60±5.580	23.77±5.20	1.295	0.197	5.376
	Stability	8.97±2.411	9.68±1.910	1.965	0.051	2.152
	Coordination	8.03±3.092	7.64±2.658	-0.811	0.419	2.863
	Strength control	5.60±2.505	6.45±2.690	1.938	0.055	2.608
**Chinese Character Writing**						
**Pause duration (s)**		61.68±33.915	51.65±19.767	2.203	0.029*	27.120
**Write velocity (pcs/s)**		0.076±0.029	0.078±0.027	0.558	0.577	0.027

**Performance**					
Completeness	9.00(2.00)	10.00(1.00)	2046	0.030*	-0.183
Penmanship	3.00(7.00)	8.00(7.00)	1811	0.003**	-0.253
Stroke positioning	5.00(4.00)	7.00(4.00)	1786	0.002**	-0.255
Spatial layout	4.00(5.00)	6.00(4.00)	1904	0.010*	-0.215
**Literacy-related cognitive abilities**					
**Orthographic awareness**					
OBS_Character	5.00(2.00)	5.00(2.00)	2154	0.083	-0.155
Pic_Character	3.00(3.00)	5.00(3.00)	2247	0.212	-0.145
Awareness Development	4.00(0.00)	4.00(1.00)	2115	0.065	-0.104
**RAN (s)**	18.70±5.421	17.50±4.457	-1.45	0.149	4.918
**Motor**					
**Pencil grip**	4.00(2.00)	4.00(2.00)	2423.000	0.601	-0.044

**The other assessments**					
**Writing pressure**					
Line drawing task	7637.23±494.816	7617.51±552.80	-0.223	0.824	527.272
Word copying task	6761.60±865.376	7021.81±783.565	1.886	0.061	821.709
**Pinch strength (N)**					
Tip pinch	25.04±7.239	21.53±5.340	-1.442	0.152	6.274
Palmar pinch	17.74±5.788	15.89±4.220	-2.205	0.029	4.993
Key pinch	17.18±5.545	15.94±4.776	-3.332	0.001	5.140

***Note.* *p<0.05.** Sensitivity analysis using non-parametric tests yielded consistent results. Cohen's d was calculated as the effect size for t-tests, and r was calculated for Mann-Whitney U tests. Significance levels were adjusted using the Bonferroni correction α= 0.0031 (0.05/16). Indices that reached this threshold are marked with **.

).

Appendix Table C.3. Distributional Characteristics and Ceiling Effects of CPAT

Items	Frequency of max score	Percentage of max score (%)	CV (%)
**Visual-Motor Integration (VMI)**			
**Pause duration**	/	/	54.29
**Route velocity**	/	/	41.97
**Performance**			
Stability	28	19.58	23.18
Coordination	18	12.59	36.57
Strength control	0	0.00	43.40
**Chinese Character Writing**			
**Pause duration**	/	/	48.93
**Write velocity**	/	/	35.63
**Performance**			
Completeness	77	53.85	14.25
Penmanship	41	28.67	66.84
Stroke positioning	15	10.49	50.62
Spatial layout	7	4.90	56.89
**Literacy-related cognitive abilities**			
**Orthographic awareness**			
OBS_Character	84	58.74	35.70
Pic_Character	70	48.95	50.87
Awareness Development	38	26.57	26.55
**RAN**	/	/	27.44
**Motor**			
**Pencil grip**	86	60.14	29.02

Note. CV: Coefficient of Variation = SD/mean

Appendix Table D.1. Test-retest reliability for the VMI and RAN

	ICC	SD_{pooled}	SEM	MDC
VMI-Pause Time	0.842	9.900	3.935	10.908
VMI-Route Velocity	0.891	0.112	0.037	0.103
VMI-Performance	0.861	6.111	2.278	6.315
RAN	0.810	5.535	2.413	6.688

Note. ICC: Intraclass Correlation Coefficients; SEM: Standard Error of Measurement; MDC: Minimal Detectable Change

Appendix Table D.2. Rater reliability for the CPAT

**Item**	**Intraclass Correlation Coefficient, ICC**	**p value**
**Visual-Motor Integration (VMI)- Line drawing task**		
Stability	0.829	<0.001
Coordination	0.908	<0.001
Strength control	0.873	<0.001
Performance	0.94	<0.001
**Writing- Word copying task**		
Completeness	0.918	<0.001
Penmanship	0.889	<0.001
Stroke position	0.901	<0.001
Spatial layout	0.941	<0.001
Performance	0.958	<0.001

Appendix Table E. Validation of content validity of CPAT

Items	Expert Ratings					Experts in Agreement	Item I-CVI	Mark
	A	B	C	D	E			
**Visual-Motor Integration (VMI)**								
**Pause duration**	4	4	4	4	4	5	1.00	Excellent
**Route velocity**	4	4	4	4	4	5	1.00	Excellent
**Accuracy performance**								
Stability	4	4	4	4	4	5	1.00	Excellent
Coordination	4	4	4	4	4	5	1.00	Excellent
Strength control	4	4	4	4	4	5	1.00	Excellent
**Chinese Character Writing**								
**Pause duration**	4	4	4	4	4	5	1.00	Excellent
**Write velocity**	4	4	4	4	4	5	1.00	Excellent
**Legibility Performance**								
Completeness	4	4	4	4	4	5	1.00	Excellent
Penmanship	4	4	4	4	4	5	1.00	Excellent

Stroke positioning	4	4	4	4	4	5	1.00	Excellent
Spatial layout	4	4	4	4	4	5	1.00	Excellent
**Literacy-related cognitive abilities**								
**Orthographic awareness**								
OBS_Character	4	4	4	4	4	5	1.00	Excellent
Pic_Character	4	4	4	4	4	5	1.00	Excellent
Awareness Development	4	4	4	4	4	5	1.00	Excellent
**RAN**	4	4	4	4	4	5	1.00	Excellent
**Motor**								
Pencil grip	4	4	4	4	4	5	1.00	Excellent
						**Average I-CVI=**	**1.00**	
**Proportion Relevant**	1.00	1.00	1.00	1.00	1.00			

The table shows the result of the five expert ratings of the CPAT, items rated 3 or 4 on a 4-point Lister scale. A, B, C, D and E represent one expert each I-CVI, item-level content validity index; Scale-level content validity index, Universal agreement method (S-CVI/UA)= 1.00; Scale-level content validity index, averaging method (I-CVI/Ave)= 1.00 ; average proportion of items judged relevant across the three experts= 1.00.

Appendix Table F.1. Factor Loadings from the Exploratory Factor Analysis of the CPAT (*N*=-143)

Item	Factor 1-Writing Performance	Factor 2-Orthographic Awareness	Factor 3-Writing Fluency	h^2
Writing- Stroke positioning	**0.867**			0.744
Writing- Penmanship	**0.817**			0.646
Writing- Spatial layout	**0.723**			0.572
Writing- Completeness	**0.545**			0.448
VMI- Performance	0.368			0.173
Pencil grip	0.261			0.066
OA- OBS_Character		**0.858**		0.751
OA- Pic_Character		**0.776**		0.620
OA- Awareness Development		**0.773**		0.571
RAN		0.170		0.116
Writing- Write velocity			**0.840**	0.725
Writing- Pause Time			**0.629**	0.532
VMI- Route velocity			**0.554**	0.276
VMI- Pause Time			0.339	0.114
**Eigenvalues**	2.60	2.08	1.67	
**Variance Explained (%)**	18.6	14.9	11.9	
**Cumulative Variance (%)**	18.6	33.5	45.4	

Note. Factor loadings > .40 are bolded. h^2 = Communality. Extraction Method: Minimum Residual. Rotation Method: Oblique. Model Fit: $\chi^2(91) = 689.00$, $p < .001$; TLI = 0.941; RMSEA = 0.051 (90% CI: 0.014–0.079).

Appendix Table F.2 Discriminant validity of Model 1

	Writing Performance	Writing Fluency	Orthographic awareness
**Writing Fluency**	0.39***		
**Orthographic awareness**	0.43***	0.40***	
**Square root of AVE**	**0.77**	**0.78**	**0.80**

Note. **** $p<.001$

Appendix Figure G. The SEM of with pencil grip as an intermediate variable.

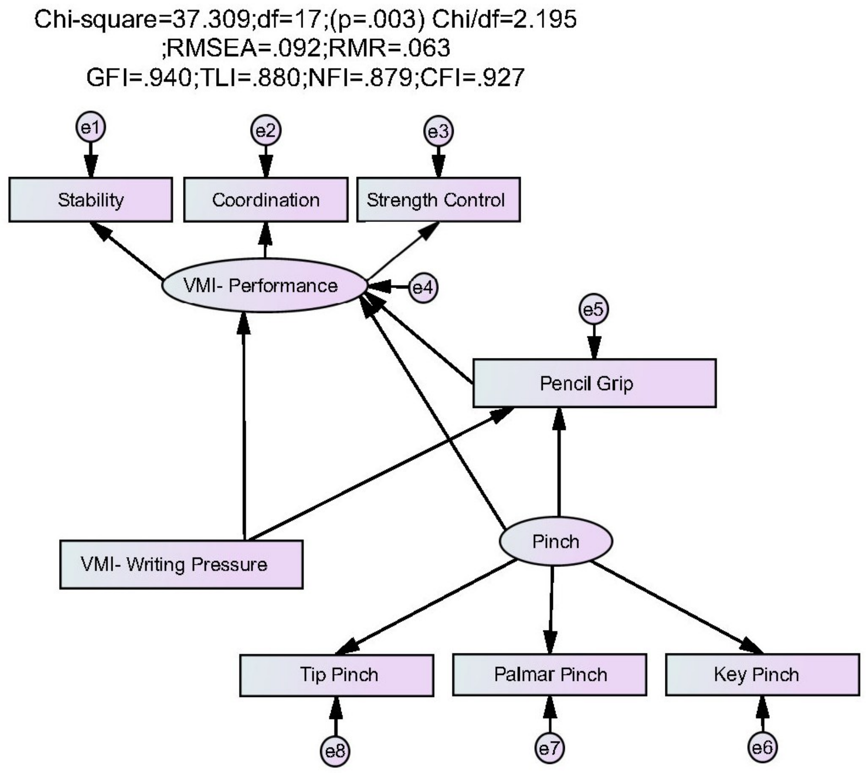

Appendix Table H. Comparison of CPAT Performance Scores Across Three SES Levels.

Items (Raw score)		High SES (*n*=84)	Middle SES (*n*=35)	Low SES (*n*=24)	*H*	*P* value
**Visual-Motor Integration (VMI)- Line drawing task**						
**Pause duration (s)**		37.17(19.58)	42.46(21.77)	36.83(21.19)	1.714	0.4245
**Trajectory velocity (cm/s)**		0.78(0.36)	0.61(0.20)	0.64(0.27)	10.006	0.0067
**Performance**						
	Stability	10.00(3.00)	10.50(2.75)	9.00(2.50)	5.224	0.073
	Coordination	7.00(3.00)	9.00(2.75)	8.50(4.00)	6.124	0.047
	Strength control	5.00(5.00)	8.00(5.75)	7.50(4.25)	4.41	0.11
**Writing- Word copying task**						
**Pause duration (s)**		48.59(25.27)	51.66(38.91)	47.35(36.08)	0.001	0.9996
**Write velocity (pcs/s)**		0.07(0.03)	0.07(0.04)	0.07(0.04)	0.159	0.9236
**Performance**						
	Completeness	9.50(2.00)	10.00(1.00)	9.50(1.00)	3.433	0.18
	Penmanship	5.00(7.25)	9.00(7.50)	9.00(5.50)	3.311	0.191
	Stroke position	5.50(5.00)	6.00(4.00)	6.00(4.00)	0.704	0.703

Spatial layout	5.00(5.25)	6.00(4.00)	5.00(5.00)	1.258	0.533
**Literacy-related cognitive abilities**					
**Orthographic awareness**					
OBS_Character	5.00(3.00)	5.00(0.50)	5.00(2.00)	6.645	0.036
Pic_Character	3.00(3.00)	5.00(2.00)	4.50(3.00)	5.738	0.057
Awareness Development	4.00(2.00)	4.00(1.00)	4.00(0.25)	1.316	0.5178
**RAN (s)**	17.76(5.70)	16.92(5.09)	17.76(5.16)	0.645	0.7244
**Motor**					
**Pencil grip**	4.00(2.00)	4.00(1.50)	4.00(2.00)	1.414	0.493

Appendix Figure I. Chinese writing performance of children with different pencil grips.

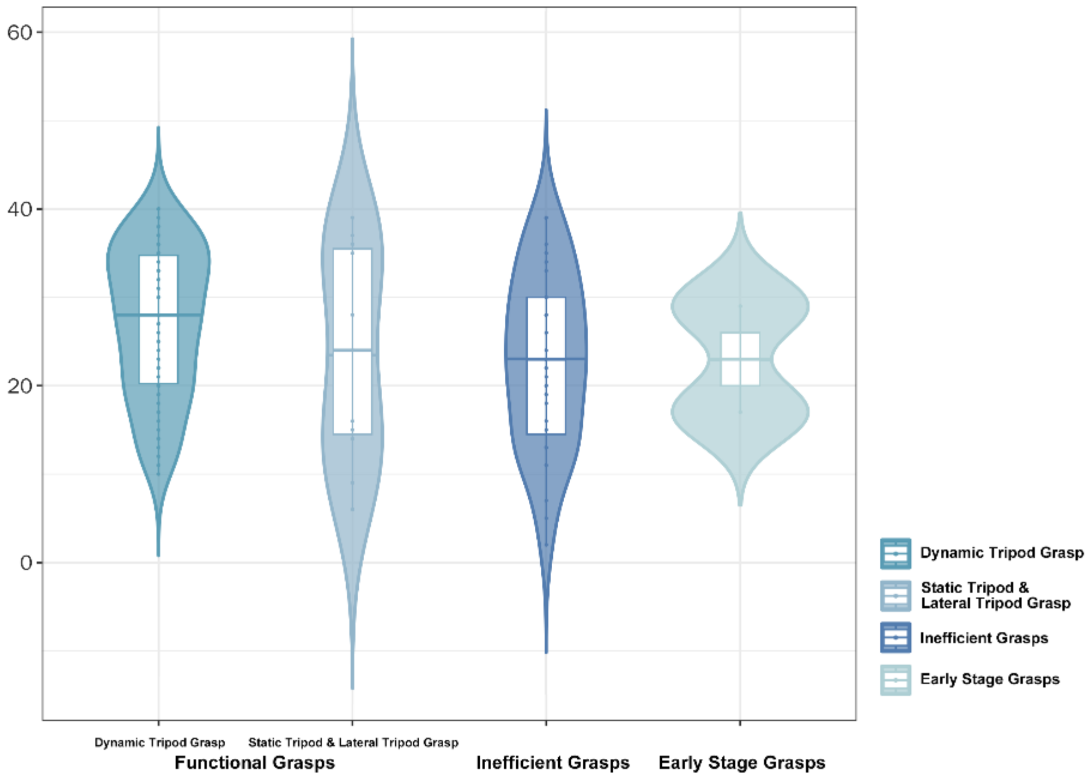

Supplement: Supplementary file 1 [file Supplementary_file_1.pdf]
